# Isolation and Cs^+^ resistance mechanism of *Escherichia coli* strain ZX-1

**DOI:** 10.3389/fmicb.2023.1340033

**Published:** 2024-01-18

**Authors:** Daiki Kojima, Shunsuke Tanaka, Ayane Kurosaki, Xiong Zhiyu, Masahiro Ito

**Affiliations:** ^1^Graduate School of Life Sciences, Toyo University, Oura-gun, Gunma, Japan; ^2^Faculty of Life Sciences, Toyo University, Oura-gun, Gunma, Japan; ^3^Bio-Nano Electronics Research Center, Toyo University, Kawagoe, Saitama, Japan; ^4^Bio-Resilience Research Project (BRRP), Toyo University, Oura-gun, Gunma, Japan

**Keywords:** cesium-resistance mechanism, *Escherichia coli*, mutant, whole-genome sequencing, ribosome

## Abstract

This research aims to elucidate the physiological mechanisms behind the accidental acquisition of high-concentration cesium ions (Cs^+^) tolerance of *Escherichia coli* and apply this understanding to develop bioremediation technologies. Bacterial Cs^+^ resistance has attracted attention, but its physiological mechanism remains largely unknown and poorly understood. In a prior study, we identified the Cs^+^/H^+^ antiporter TS_CshA in *Microbacterium* sp. TS-1, resistant to high Cs^+^ concentrations, exhibits a low Cs^+^ affinity with a *K*_m_ value of 370 mM at pH 8.5. To enhance bioremediation efficacy, we conducted random mutagenesis of *TS_cshA* using Error-Prone PCR, aiming for higher-affinity mutants. The mutations were inserted downstream of the P_BAD_ promoter in the pBAD24 vector, creating a mutant library. This was then transformed into *E. coli*-competent cells. As a result, we obtained a Cs^+^-resistant strain, ZX-1, capable of thriving in 400 mM CsCl—a concentration too high for ordinary *E. coli*. Unlike the parent strain Mach1^™^, which struggled in 300 mM CsCl, ZX-1 showed robust growth even in 700 mM CsCl. After 700 mM CsCl treatment, the 70S ribosome of Mach1^™^ collapsed, whereas ZX-1 and its derivative ΔZX-1/pBR322ΔAp remained stable. This means that the ribosomes of ZX-1 are more stable to high Cs^+^. The inverted membrane vesicles from strain ZX-1 showed an apparent *K*_m_ value of 28.7 mM (pH 8.5) for Cs^+^/H^+^ antiport activity, indicating an approximately 12.9-fold increase in Cs^+^ affinity. Remarkably, the entire plasmid isolated from ZX-1, including the *TS_cshA* region, was mutation-free. Subsequent whole-genome analysis of ZX-1 identified multiple SNPs on the chromosome that differed from those in the parent strain. No mutations in transporter-related genes were identified in ZX-1. However, three mutations emerged as significant: genes encoding the ribosomal bS6 modification enzyme RimK, the phage lysis regulatory protein LysB, and the flagellar base component protein FlgG. These mutations are hypothesized to affect post-translational modifications, influencing the *K*_m_ value of TS_CshA and accessory protein expression. This study unveils a novel Cs^+^ resistance mechanism in ZX-1, enhancing our understanding of Cs^+^ resistance and paving the way for developing technology to recover radioactive Cs^+^ from water using TS_CshA-expressing inverted membrane vesicles.

## 1 Introduction

Cesium exposure garnered worldwide attention following the Fukushima power plant accident in 2011 and the subsequent release of substantial amounts of radioactive cesium isotopes, ^134^Cs and ^137^Cs (Hirose, 2016; Nakamura et al., 2022; Wu et al., 2022). The latter part of the 2010s saw a surge in research focusing on radioactive Cs contamination of soil, exploring decontamination strategies and bioremediation approaches, which included identifying microorganisms with high resistance to Cs^+^. Given that Cs^+^ shares chemical properties with potassium ions (K^+^), it enters microorganisms, animal cells, and plant cells through the K^+^ uptake system and inhibits growth (Hampton et al., 2004; Kato et al., 2016).

**Figure 1:**
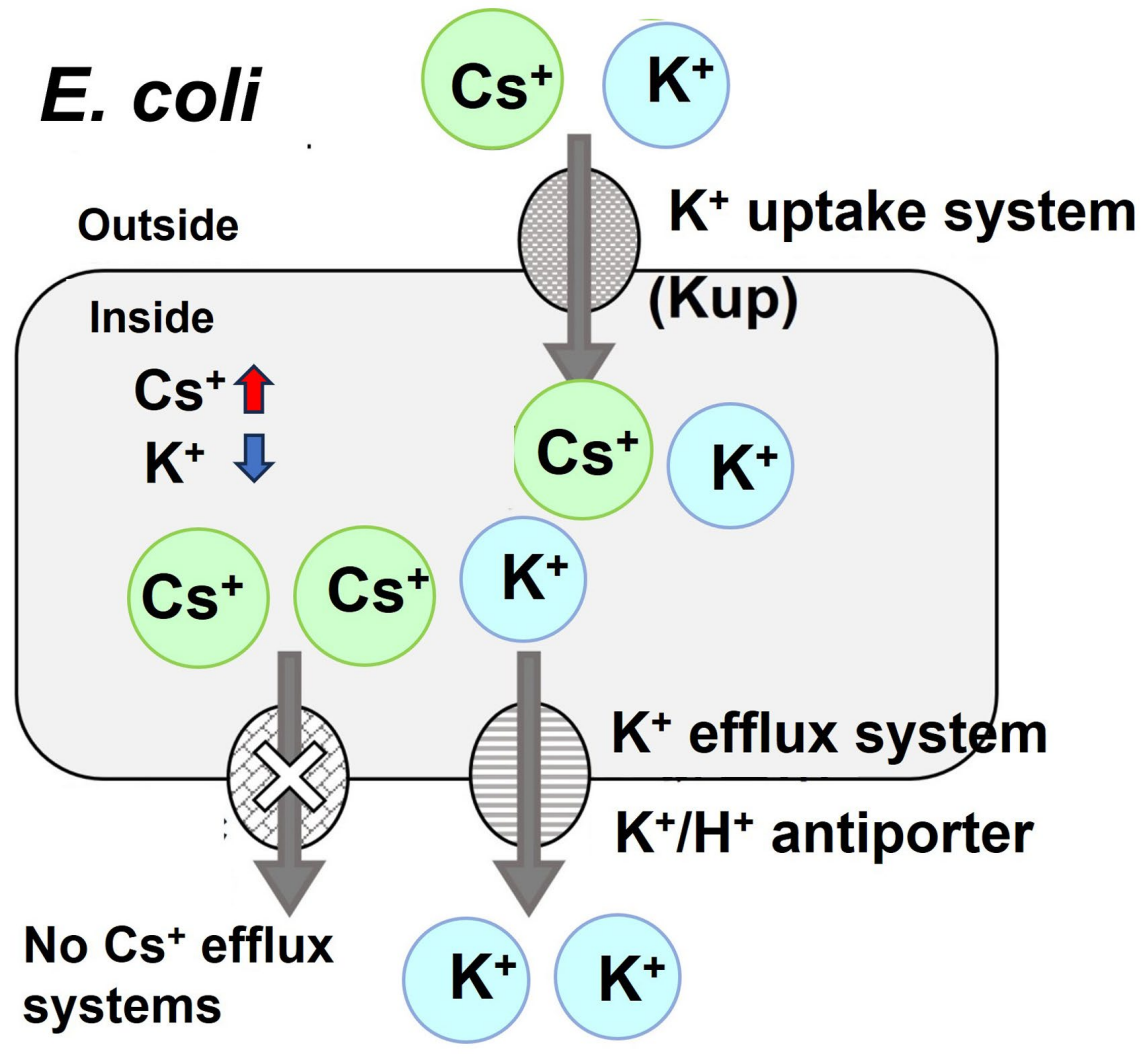
Schematic diagram of the toxicity of cesium ions to *E. coli* growth. Cesium is chemically similar to potassium, which means that it can be taken up by the potassium uptake system of *E. coli*, potentially leading to its accumulation inside the cells. Although *E. coli* expels K^+^, it lacks a specific mechanism for the efflux of Cs^+^, resulting in the buildup of Cs^+^ and a concurrent decrease in K^+^ concentration within the cell. This inhibits the growth of *E. coli*.

*Escherichia coli* cytotoxicity against Cs^+^ can be attributed to the incorrect influx of Cs^+^ via the K^+^ uptake system and the lack of a Cs^+^ excretion mechanism, leading to elevated intracellular Cs^+^ concentrations. Furthermore, higher intracellular Cs^+^ concentrations lead to the expulsion of intracellular K^+^ through the K^+^ excretion system to maintain vital homeostatic processes, such as intracellular turgor pressure, resulting in a drastic decrease in intracellular K^+^ concentration, resulting in growth inhibition (see Figure 1; Bossemeyer et al., 1989). Our group recently demonstrated that even in *Bacillus subtilis*, a gram-positive bacterium, the intracellular K^+^ concentration dramatically decreases when exposed to high Cs^+^ concentrations (Ishida et al., 2023b).

Since 2014, several studies have isolated bacteria with notable resistance to elevated Cs^+^ concentrations. Numerous microorganisms showing Cs^+^ resistance have been identified (Bossemeyer et al., 1989; Buesseler et al., 2012; Kato et al., 2016; Swer et al., 2016). Notably, *Rhodococcus qingshengii* CS98, *Arthrobacter* sp. KMSZP6, which is recognized for its Cs-accumulating capacity, has been used for bioremediation in environments polluted with radioactive Cs^+^ (Takei et al., 2014; Swer et al., 2016). *Flavobacterium* sp. 200CS-4 (Kato et al., 2016), *Serratia* sp. Cs60-2 (Dekker et al., 2014), *Yersinia* sp. Cs67-2 (Dekker et al., 2014), and *Bacillus* sp. C-700 (Zhang et al., 2021) exhibits resistance to CsCl at concentrations of 200, 300, 500, and 700 mM. *Flavobacterium* sp. 200CS-4, isolated from forest soil in Hokkaido, demonstrated a lower intracellular Cs^+^ concentration than that in the surrounding environment, although the exact resistance mechanism remains unclear (Kato et al., 2016). Similarly, *Serratia* sp. Cs60-2, and *Yersinia* sp. Cs67-2, isolated from a nuclear fuel reservoir in Cambria, USA, exhibits Cs^+^ resistance that is not observed in related species (Dekker et al., 2014). Although these bacteria display compelling Cs^+^ resistance properties, the mechanisms underlying this resistance remain uncharacterized. *Arthrobacter* sp. KMSZP6, identified from an untouched uranium deposit in India, exhibited a fascinating characteristic: when exposed to a Cs^+^-enriched solution, it not only accumulated Cs^+^ within its cells but also had a dry weight nearly triple that of its pre-exposure state (Swer et al., 2016). Therefore, *Arthrobacter* spp. KMSZP6 holds potential for bioremediation tasks such as purifying Cs^+^-contaminated water, although the cellular mechanisms underlying Cs^+^ accumulation and resistance remain unclear. Finally, *Bacillus* sp. Cs-700, extracted from sediments in the South China Sea and identified through 16S rRNA and whole-genome analyses, maintains its Cs^+^ resistance mechanism, which is shrouded in mystery (Zhang et al., 2021). In summary, while there have been multiple instances of identifying Cs^+^-resistant bacteria, the mechanisms and proteins contributing to Cs^+^ resistance remain largely unclear.

Remarkably, our team was the first to identify a gene associated with Cs^+^ resistance, named *TS_cshA* (Koretsune et al., 2022). TS_CshA, a 12-transmembrane protein transporter, belongs to the Major Facilitator Superfamily and is recognized as the most extensive family of membrane transporters. Through enzyme activity assays, TS_CshA was identified as a Cs^+^/H^+^ antiporter characterized by its low affinity (with an apparent *K*_m_ value of approximately 370 mM at pH 8.5) for extruding Cs^+^ as a substrate (Koretsune et al., 2022). In this study, we aimed to obtain a mutant TS_CshA with an augmented affinity. Random mutations were introduced into the *TS_cshA* region using Error-Prone PCR and subsequently ligated to the pBAD24 vector, which carries an arabinose-inducible promoter (Koretsune et al., 2022). To develop a TS_CshA variant with enhanced affinity for Cs^+^, we transformed *E. coli* Mach1™ (Invitrogen) competent cells. Intriguingly, although our primary goal was to increase TS_CshA affinity for Cs^+^, the *E. coli* host cells unexpectedly exhibited resistance to higher concentrations of Cs^+^. This serendipitous discovery prompted us to investigate the underlying mechanism of the newly discovered Cs^+^ resistance in the *E. coli* variant.

## 2 Materials and methods

### 2.1 Bacterial strains and plasmids

The bacterial strains and plasmids used in this study are detailed in Table 1, while a brief overview of each transformant is presented in Table 2. The primers used are available upon request. The pBR322ΔAp plasmid was obtained by digesting pBR322 with ScaI and SspI, which removed the 3′ end of the ampicillin resistance gene. The digested DNA fragments were re-ligated using T4 DNA Ligase to construct pBR322ΔAp. Consequently, this plasmid retains the same replication machinery as pBAD24 and encodes the tetracycline resistance gene.

**Table 1:** Bacterial strains and plasmids used in this study.

Strain	Genotype	References
Mach1^™^	F^−^, [φ80*lac*ZΔM15], Δ*lac*X74, *hsd*R, (r_K_^−^, m_K_^+^), Δ*rec*A1398, *end*A1, *ton*A	Thermo Fisher
ZX-1	Strain ZX-1 carrying pBAD_CshA, Ap^R^.	This study
ΔZX-1	Strain ΔZX-1 carrying pBR322ΔAp, Tet^R^, Plasmid pBAD_CshA were cured from strain ZX-1 by using incompatibility removal	This study
Plasmids		
pBAD24	Cloning expression vector, P_BAD_ promoter, Ap^R^	Guzman et al. (1995)
pBAD_CshA	pBAD24 + *TS_cshA*, Ap^R^, the former name was pBAD-00475.	Koretsune et al. (2022)
pBR322ΔAp	Cloning vector, Tet^R^	This study

**Table 2:** Brief characteristics of each transformant used in this study.

Strain	Plasmid	Description
Mach1^™^	pBAD24 (Vector)	Mach1^™^ carrying cloning vector pBAD24
Mach1^™^	pBAD_CshA	Mach1^™^ carrying pBAD_CshA
ZX-1	pBAD_CshA	High-concentration Cs^+^ resistant bacterium derived from Mach1^™^ /pBAD_CshA. pBAD_CshA is mutation-free.
ΔZX-1	pBR322ΔAp	A transformant in which pBR322ΔAp was introduced into ZX-1/pBAD_CshA resulted in the exclusion of pBAD_CshA, selected based on Tetracycline resistance due to incompatibility.
ΔZX-1	pBAD_CshA	A transformant in which pBAD_CshA was introduced into ΔZX-1/pBR322ΔAp resulted in the exclusion of pBR322ΔAp, selected based on Ampicillin resistance due to incompatibility.

### 2.2 Growth media and conditions

*Escherichia coli* strains were grown at 37°C in Luria-Bertani (LB) medium (BD Difco^™^, New Jersey, USA) with different concentrations of Cesium Chloride. *E. coli* Mach1^™^ (Thermo Fisher) and its derivatives were used in this study for routine genetic manipulation and antiport assays. The medium was supplemented with Tetracycline (25 μg/mL) or ampicillin (100 μg/mL) when antibiotics were required for growth selection. Cells were grown at 37°C with shaking and monitored by measuring the optical density at 600 nm (OD_600_) using a UV-1800 ultraviolet–visible spectrophotometer (Shimadzu Co., Ltd., Kyoto, Japan).

### 2.3 TS_CshA mutation induction by error-prone PCR and preparation of the CshA insertion vector

The TS_CshA mutation was created through Error-Prone PCR using the Diversify^™^ PCR Random Mutagenesis Kit (Takara Bio, Japan). The primers pBAD-NheI-F (AAG ATT AGC GGA TCC TAC CTG) (5′ → 3′) and pBAD-XbaI-F (CGG ATG AGA GAA GAT TTT CAG) (5′ → 3′) were used. PCR was performed according to the manufacturer’s instructions, with conditions producing approximately eight nucleotide substitutions per kilobase. The PCR products were purified using a QIAprep Gel Extraction Kit (Qiagen, Netherlands). The constructs were assembled using NEBuilder HiFi DNA Assembly Master Mix (NEB, United States). Error-prone PCR reactions were independently executed three times. After each instance, colonies that emerged were randomly selected, and their DNA sequences were analyzed to ascertain if mutations had been introduced into the *cshA* gene. In a previous study, plasmid pBAD24, which cloned the *TS_cshA* gene region, was referred to as pBAD-00475 (Koretsune et al., 2022). However, for clarity, it was renamed pBAD_CshA in this paper.

pBAD_CshA was prepared and transformed into *E. coli* Mach1^™^. The replica plating method was used to screen for Cs^+^-resistant strains in the transformed *E. coli*. Initially, *E. coli* cells were plated on LB agar, and the resulting colonies were replicated on fresh LB agar plates containing varying concentrations of CsCl (0, 200, 300, and 400 mM). Strains capable of growing in a medium containing high CsCl concentrations were selected.

### 2.4 Cs^+^ resistance growth assay

Each *E. coli* transformant was pre-cultured in LB medium for 5 h. Subsequently, 10 μL of pre-cultured cells were added to 2 mL LB medium containing 0 to 1,000 mM CsCl at 100 mM intervals and incubated for 18 h. After incubation, the turbidity of the cultures was measured at 600 nm using a UV-1800 ultraviolet–visible spectrophotometer (Shimadzu Co., Ltd., Kyoto, Japan).

In addition, in order to evaluate the effect on the growth of each transformed *E. coli* in the presence of Cs^+^, each *E. coli* was precultured for 18 h in 5 mL of LB medium, and the CsCl concentration was 0 mM, 200 mM, and 700 mM, respectively. The culture was started by adding 500 μL of the preculture solution to a 500 mL fluted Erlenmeyer flask containing 100 mL of LB medium. The turbidity of the culture solution at OD_600_ was measured at 1-h intervals from 0 h to 8 h.

### 2.5 Measurement of Cs^+^/H^+^ antiport activity in the everted membrane vesicles for transforming *Escherichia coli*

Inverted membrane vesicles from strains Mach1^™^ /pBAD24, ZX-1, ΔZX-1/pBR322ΔAp, and ΔZX-1/pBAD_CshA were prepared using the standard method described by Nozaki et al. (1998) to assay Cs^+^/H^+^ antiport activity. First, each *E. coli* strain was cultured and then collected by centrifugation at 10,740 × *g* and 4°C for 15 min. Each pellet was washed with 25 mL of TCDG Buffer (10 mM Tris–HCl, pH 8.0, 5 mM MgCl_2_, 10% glycerol, 140 mM choline chloride, and 1 mM D-dithiothreitol). Afterward, each of them was resuspended in 25 mL of the same buffer to which half a tablet of EDTA-free protease inhibitor (Roche), half a spatula of DNase I (Roche) and 250 μL of 0.1 M phenylmethanesulfonylfluoride were added. The suspensions were subjected to two passes through a French press at 10,000 psi. The supernatants were collected after centrifugation at 9,100 × *g* at 4°C for 10 min. The supernatants were then ultracentrifuged at 49,000 × *g*, at 4°C, for 1 h to pellet the inverted membrane vesicle fraction. The fraction was homogenized in 1 mL of TCDG buffer and stored at −80°C. Protein concentrations were determined using the Lowry method (Lowry et al., 1951) with bovine serum albumin (BSA) as the standard.

The Cs^+^/H^+^ antiport activity was measured using the fluorescence quenching method of Swartz et al. (2007). with a model F-4500 fluorescence spectrophotometer (Hitachi High-Technologies, Japan) at an emission wavelength at 420 nm, emission wavelength at 500 nm, and slit width of 10 nm. Initially, a stirrer was placed in a four-sided cuvette, and the assay buffer (2 mL, 140 mM choline chloride, 50 mM Bis-Tris propane, pH adjusted to 8.5 with H_2_SO_4_), 0.7 μL of 1 mM acridine orange, and 66 μg of inverted membrane vesicles were added to commence the measurement. After baseline fluorescence intensity stabilized, 5 μL of 1 M succinate was added to trigger respiration. The uptake of succinate by the inverted membrane vesicles lowers the intracellular pH, which in turn causes acridine orange to enter the vesicles, markedly reducing fluorescence intensity. Once the decrease in fluorescence stabilized, an arbitrary concentration of 2 M Cs_2_SO_4_ was added to assess antiport activity. If present, Cs^+^ uptake and H^+^ expulsion would lead to acridine orange migration out of the vesicles, thereby increasing the fluorescence intensity. After this intensity stabilized, 5 μL of 4 M NH_4_Cl was added to reset the fluorescence to baseline levels. The measurements were concluded when the fluorescence intensity returned to a stable state.

The percentage of fluorescence quenching was calculated relative to the initial succinate-induced quenching and the subsequent increase (de-quenching) upon Cs^+^ addition.

A Lineweaver-Burk plot was generated from the dequenching percentages and Cs^+^ concentrations to determine the apparent *K_m_* value and examine the Cs^+^ substrate affinity of each transformed *E. coli* strain.

### 2.6 Measuring intracellular Cs^+^ and K^+^ concentrations

Samples were prepared as described by Ito et al. (1997). Assays to determine intracellular Cs^+^ and K^+^ concentrations were performed on strains ZX-1, ΔZX-1/pBR322ΔAp, Mach1/pBAD24, and Mach1/pBAD_CshA. Strains ZX-1 and ΔZX-1/pBR322ΔAp were grown under conditions of 0 mM, 200 mM, and 700 mM CsCl. Mach1/pBAD24 and Mach1/pBAD_CshA cells were cultured in the presence of 0 and 200 mM CsCl, respectively. The strains were grown to an OD_600_ of 0.4, followed by centrifugation at 5,800 *× g* at 4°C for 3 min to collect the cells. The cell pellet was washed twice with 5 mL of 300 mM sucrose. During the second wash, 100 μL of the suspension was taken to assess protein concentration using the Lowry method. To the remaining pellet, 5 mL of 5% trichloroacetic acid was added, and the sample was heat-treated at 100°C for 10 min. After heat treatment, the sample was centrifuged at 9,100 *× g* at 4°C for 10 min to separate the supernatant. The Cs^+^ and K^+^ concentrations in the supernatant were measured using a flame photometer (OSK55XC750-PLUS) (Ogawa Seiki, Japan). The Cs^+^ and K^+^ standards were prepared by diluting a standard solution of 1,000 mg/L. Intracellular ion concentrations were calculated based on a cell volume equivalent to 3 μL per 1 mg of protein.

### 2.7 Whole plasmid sequencing

Plasmid pBAD_CshA derived from Mach1^™^ and ZX-1 was prepared according to the manufacturer’s instructions. The complete nucleotide sequences of the plasmids were determined through full-length sequencing and annotation of circular plasmid DNA using long-read sequencing technology provided by Oxford Nanopore Technologies (ONT).

### 2.8 Whole-genome sequencing

A single colony of ZX-1 was inoculated into 2 mL of LB medium to prepare a pre-culture, which was then incubated with shaking at 200 rpm at 37°C for 8 h. Subsequently, 500 μL of this pre-culture was transferred into a 24φ test tube containing 4.5 mL of LB medium. This mixture was further incubated at 37°C with reciprocal shaking at 200 rpm for 4 h. The entire culture was centrifuged at 9,100 *× g* at 4°C for 5 min, and the supernatant was discarded. Genomic DNA was extracted using the DNeasy Blood and Tissue Kit (QIAGEN, Hilden, Germany) according to the manufacturer’s instructions.

Whole-genome sequencing and subsequent comparative analyses were performed by Eurofins Genomics, Inc. using a next-generation sequencing platform (HiSeq X, 2 × 150 bp, Illumina, USA). The whole-genome sequences of strains ZX-1 and Mach1^™^ were scrutinized for single nucleotide polymorphisms (SNPs), and mutation sites were identified. The SNP analysis was conducted using the following workflow: SAMtools (ver. 1.6) was used to identify bases that differed from the reference sequences in the mapped results. Variants that met the predefined criteria were extracted using vcfutils.pl. from bcftools (ver. 1.6). Genes with mutation sites were identified as potential candidates for Cs^+^ resistance. The sequence data generated are available in the DNA Data Bank of Japan (DDBJ) Sequence Read Archive under accession numbers DRA017249 (ZX-1) and DRA017250 (Mach1^™^).

### 2.9 The preparation of ribosome

Ribosomes were prepared and fractionated using the method of Ishida et al. (2023a). Mach1^™^/pBAD24, ZX-1, and ΔZX-1/pBR322ΔAp were pre-cultured at 37°C for 16 h on an LB agar plate. Cells were collected from the colonies on the plate using an inoculation loop. These cells were then inoculated into 400 mL of LB medium at an initial OD_600_ of 0.03. The culture was incubated at 37°C and 200 rpm. When the OD_600_ reached 0.4, CsCl was added to one of the cultures, resulting in a final concentration of 700 mM. The culture was further incubated for an additional 1 or 2 h. The culture medium was centrifuged at 5000 × *g* and 4°C for 10 min using an NA-600C rotor (TOMY SEIKO Co., Ltd., Tokyo, Japan) to recover the bacteria. Cultures were collected in 20 mL of Buffer I (20 mM Tris base, 10 mM Magnesium acetate tetrahydrate, 100 mM Ammonium acetate, 0.1 mM Dithiothreitol, 2 mM phenylmethanesulfonylfluoride, and pH 7.4 (adjusted with 5 N HCl)) and breakdown in a French press (8,000 psi). The French press treatment was performed twice. Unbroken cells were removed by centrifugation at 12,000 × *g* and 4°C for 15 min to obtain a crude cell extract supernatant containing ribosomes. The absorbance of the crude cell extract was measured at 260 nm by a spectrophotometer. This experiment was performed three times to confirm reproducibility.

### 2.10 Analysis of ribosomal complexes by sucrose density gradient ultracentrifugation

An Ultra-Clear^™^ centrifuge tube (14 mL; Beckman Coulter, Brea, CA, United States) for sucrose density gradient ultracentrifugation was filled with 4.5 mL of Buffer I containing 10% sucrose. Next, a syringe was carefully filled with 4.5 mL of 35% sucrose in Buffer I into the bottom of the tube. The tubes were sealed with Parafilm and left at room temperature for 2 h in a tilted position to form a sucrose density gradient, then left at 4°C for 1 h. 1 mL of crude cell extract (10 A_260_/mL/tube) containing ribosomes was layered on top of the sucrose density gradient. Ultracentrifugation was performed at 222,000 × *g* and 4°C for 3 h using a SW40Ti rotor (6 × 14 mL; Beckman Coulter) in an Optima XPN- 100 Ultracentrifuge (Beckman Coulter). Fractions of 200 μL each were separated from the top layer of tubes (54 fractions), and A_260_ of each fraction was measured on a Thermo NanoDrop 200C (Thermo Fisher Scientific KK, Tokyo, Japan). Sucrose concentration was measured with an Atago handheld refractometer (MASTER-PM, ATAGO Co., Ltd., Tokyo, Japan).

## 3 Results

### 3.1 Attempt to obtain mutant CshA with high substrate affinity for Cs^+^ by randomly mutating TS_CshA by error-prone PCR

Previously, we reported that *Microbacterium* sp. TS-1 can thrive even in media containing 1,200 mM CsCl (Koretsune et al., 2022). The Cs^+^ resistance of TS-1 was attributed to the Cs^+^/H^+^ antiporter (TS_CshA), which effectively expelled Cs^+^ from cells. As the introduction details, *E. coli*, lacking a Cs^+^ efflux mechanism, experiences cytotoxicity. Consequently, we hypothesized that CshA expression in *E. coli* would enhance Cs^+^ resistance. However, contrary to our expectations, *E. coli* did not exhibit increased Cs^+^ resistance (Koretsune et al., 2022). This may be because the apparent *K*_m_ value of TS_CshA indicated a low substrate affinity of approximately 370 mM (pH 8.5), which might have limited the growth of *E. coli* before CshA became active. Thus, we aimed to develop a mutant CshA with higher substrate affinity by inducing random mutations in TS_CshA using Error-Prone PCR. Error-prone PCR reactions were independently executed three times. We extracted plasmids from independent colonies in three separate experiments and analyzed the number of mutated bases in each plasmid. The results are shown in Supplementary Table S1. Notably, one of the three samples (No. 1) appeared not to have undergone successful Error-prone PCR. However, using a plasmid library prepared from this sample, a ZX-1 strain capable of growing on a selection plate containing 400 mM CsCl was obtained among Mach1^™^-transformed colonies. *E. coli* Mach1^™^ transformed with the pBAD_CshA plasmid containing the mutated TS_CshA was screened using Error-Prone PCR. From approximately 50,000 colonies, a strain capable of growing in a medium containing 400 mM Cs^+^ was isolated and named strain ZX-1. However, when the pBAD_CshA plasmid from ZX-1 was introduced into the parent strain Mach1^™^, the transformants did not confer the same Cs^+^ resistance. In addition, we sequenced the pBAD_CshA plasmid from both ZX-1 (pBAD_CshA) and Mach1^™^/pBAD_CshA. The results showed that the sequences of these two plasmid DNAs were identical and mutation-free, with a 100% match (Supplementary Figure S1).

### 3.2 Cs^+^ resistance growth assay

**Figure 2:**
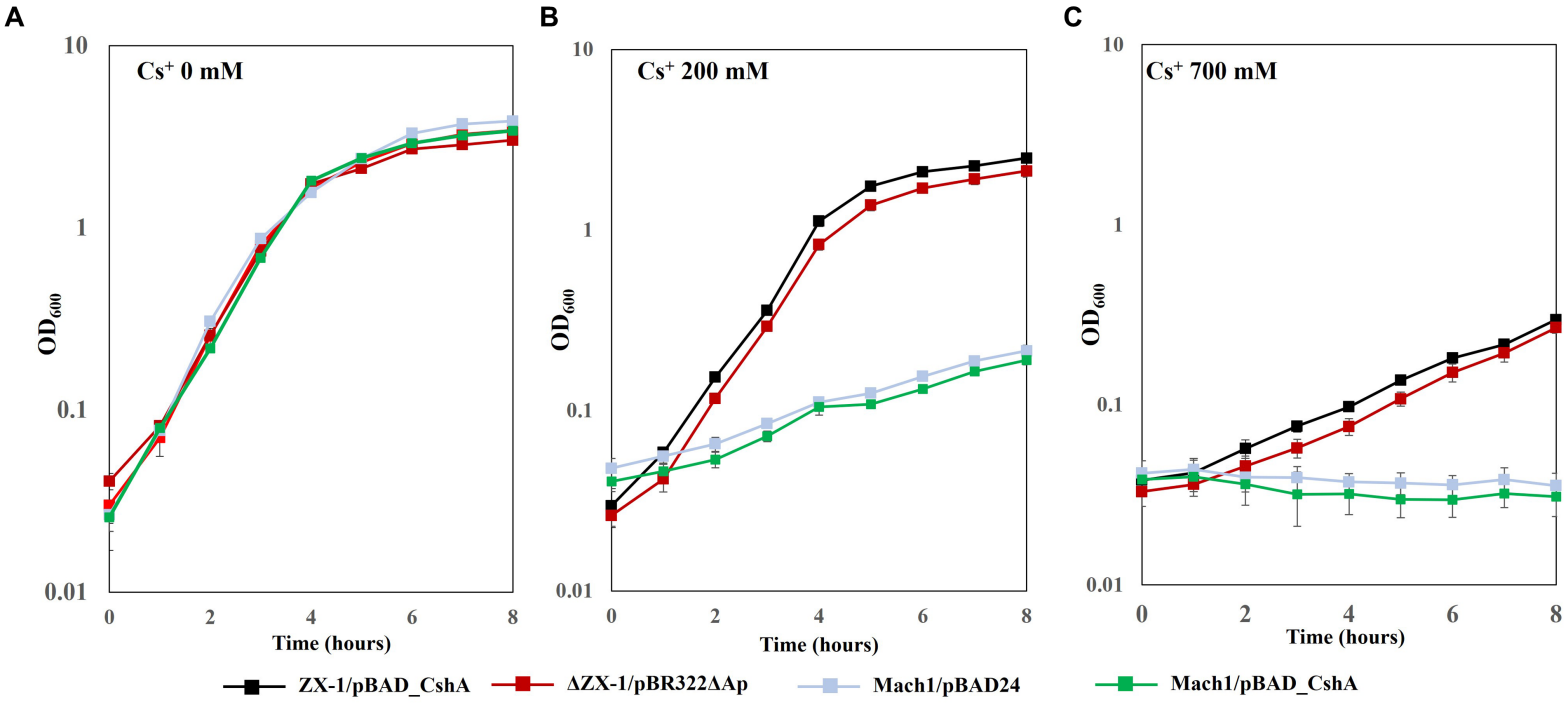
Growth curves under 0 mM, 200 mM, and 700 mM CsCl concentration conditions in each transformant of *E. coli*. Each *E. coli* strain was precultured in 5 mL of LB medium at 37°C and 200 rpm for 18 h. Subsequently, 500 μL of the preculture was inoculated into 100 mL of LB medium containing varying CsCl concentrations (0 mM **(A)**, 200 mM **(B)**, and 700 mM **(C)**), each in a separate 500 mL fluted flask. The turbidity of the culture was measured at an optical density of 600 nm (OD_600_) every hour for up to 8 h. Line graphs show strains ZX-1/pBAD_CshA (black), ΔZX-1/pBR322ΔAp (red), Mach1/pBAD24 (pale blue), and Mach1/pBAD_CshA (green). The vertical axis shows OD_600,_ and the horizontal axis shows time (hours). Error bars indicate three independent experiments.

No difference in growth rate was observed among the transformed *E. coli* strains grown on LB medium without CsCl (Figure 2A). However, under 200 mM CsCl in the LB medium, the growth rates of strains Mach1^™^/pBAD24 and Mach1^™^/pBAD_CshA, which lack Cs^+^ resistance, were significantly reduced. In contrast, the growth rates of strains ZX-1/pBAD_CshA and ΔZX-1/pBR322ΔAp remained almost unaffected (Figure 2B). In the presence of 700 mM CsCl, Mach1^™^/pBAD24 and Mach1^™^/pBAD_CshA showed no growth. Although the growth rates of strains ZX-1/pBAD_CshA and ΔZX-1/pBR322ΔAp were also reduced compared to those in 0 and 200 mM CsCl conditions, an increase in turbidity was still observed, indicating some growth (Figure 2C).

**Figure 3:**
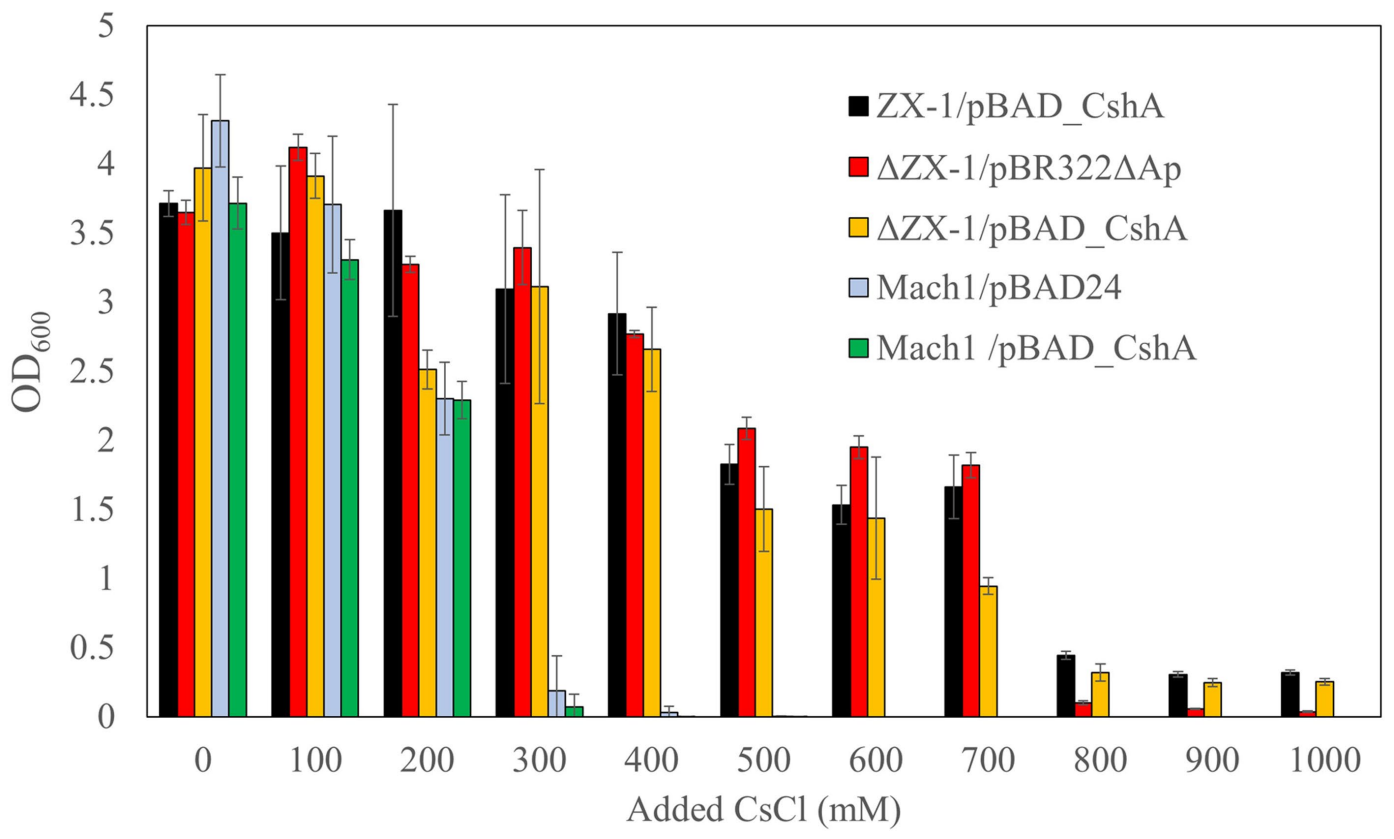
Cs^+^ resistance growth test of *E. coli* ZX-1 and its related transformants. The color coding for each bar graph is as follows: ZX-1/pBAD_CshA in black, ΔZX-1/pBR322ΔAp in red, ΔZX-1/pBAD_CshA in yellow, Mach1/pBAD24 in blue, and Mach1/pBAD_CshA in green. The vertical axis represents the optical density at 600 nm, and the horizontal axis indicates the Cs^+^ concentration. Error bars represent the standard deviation of three independent experiments.

Next, to examine the upper limit Cs^+^ concentration for the growth of each transformant, turbidity was measured after 18 h at 37°C in an LB medium with a CsCl concentration ranging from 0 to 1,000 mM (Figure 3). The growth of transformants derived from Mach1^™^, the parental strain, was inhibited at a CsCl concentration of 300 mM. Similarly, Mach1^™^ /pBAD_CshA, which Mach1^™^ was transformed with pBAD_CshA, showed growth inhibition at a CsCl concentration of 300 mM. On the other hand, the Cs^+^-resistant strain ZX-1 showed growth of >0.4 at OD_600_ in 18 h, even at 800 mM CsCl concentration. Surprisingly, despite the removal of pBAD_CshA, the strain ΔZX-1/pBR322ΔAp was able to grow at 700 mM CsCl concentration, but growth was inhibited at 800 mM CsCl concentration. When the strain ΔZX-1/pBR322ΔAp was transformed again with pBAD_CshA, it could grow at 800 mM CsCl concentration as well as the strain ZX-1.

### 3.3 Cs^+^/H^+^ antiport assay

The results of the Cs^+^ resistance test of each transformant showed that the strain ΔZX-1/pBR322ΔAp was also highly resistant to Cs^+^ even without plasmid pBAD_CshA. This led to the speculation that pBAD_CshA may not have been eliminated from strain ΔZX-1/pBR322ΔAp or that strain ZX-1 might have altered its substrate affinity for Cs^+^. Consequently, to assay CshA activity and substrate affinity for Cs^+^, we used ZX-1/pBAD_CshA, ΔZX-1/pBR322ΔAp, and ΔZX-1/pBAD_CshA.

**Figure 4:**
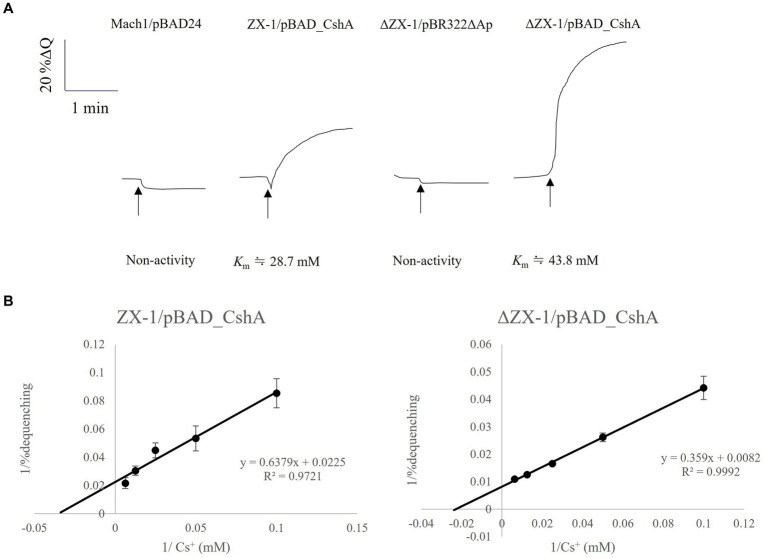
Assay of Cs^+^/H^+^ antiport activity using inverted membranes from transformed *E. coli*, with a Lineweaver-Burk plots diagram for strains ZX-1/pBAD_CshA and ΔZX-1/pBAD_CshA. **(A)** Measurements were initiated by adding 2 mL of pH 8.5 antiport activity assay buffer (50 mM bis-tris propane, 140 mM choline chloride), 0.7 μL of 1 mM acridine orange, and inverted membrane vesicle lysate equivalent to 66 μg of protein. After the fluorescence intensity stabilized, 2.5 mM succinic acid was added. After confirming that the fluorescence intensity decreased and stabilized due to respiration, 20 mM Cs_2_SO_4_ was added at the position indicated by the arrow. Traces were selected as representatives of three or more independent experiments. **(B)** Lineweaver-Burk plots were obtained from the double inverse of Cs^+^/H^+^ antiport activity at each Cs^+^ concentration for transformed *E. coli* with confirmed Cs^+^/H^+^ antiporter activity at pH 8.5. The vertical axis shows the reciprocal of the antiport activity. The horizontal axis shows the reciprocal of the Cs^+^ concentration. Error bars indicate the standard deviation of three independent experiments.

The results indicated that the strains ZX-1 and ΔZX-1/pBAD_CshA demonstrated antiport activity, as depicted in Figure 4A. Conversely, the strains ΔZX-1/pBR322ΔAp and Mach1^™^/pBAD24 exhibited no such activity. Additionally, the apparent *K*_m_ value for strain ZX-1, calculated using the Lineweaver-Burk plot equation, was 28.7 mM for Cs^+^ (Figure 4B). The apparent *K*_m_ value for Cs^+^ in the strain ΔZX-1 re-transformed with pBAD_CshA was 43.8 mM (Figure 4B). Table 3 summarizes the Cs^+^ resistance and antiport activity of the *E. coli* transformants used in this study.

**Table 3:** Growth and Cs^+^/H^+^ antiport activity in the presence of Cs^+^.

Strain	Carrying plasmid	400 mM CsCl	700 mM CsCl	Cs^+^/H^+^ antiport activity at pH 8.5
Mach1^™^	pBAD24 (vector)	Not growth	Not growth	ND ^a^
Mach1^™^	pBAD_CshA	Not growth	Not growth	Apparent *K_m_* value = 370 mM (Koretsune et al., 2022)
ZX-1	pBAD_CshA	Growth	Growth	Apparent *K_m_* value = 28.7 mM
ΔZX-1	pBR322ΔAp	Growth	Growth	ND ^a^
ΔZX-1	pBAD_CshA	Growth	Growth	Apparent *K_m_* value = 43.8 mM

^a^ Not detected.

### 3.4 Intracellular K^+^ and Cs^+^ concentrations of each *Escherichia coli* transformant under various CsCl conditions

**Figure 5:**
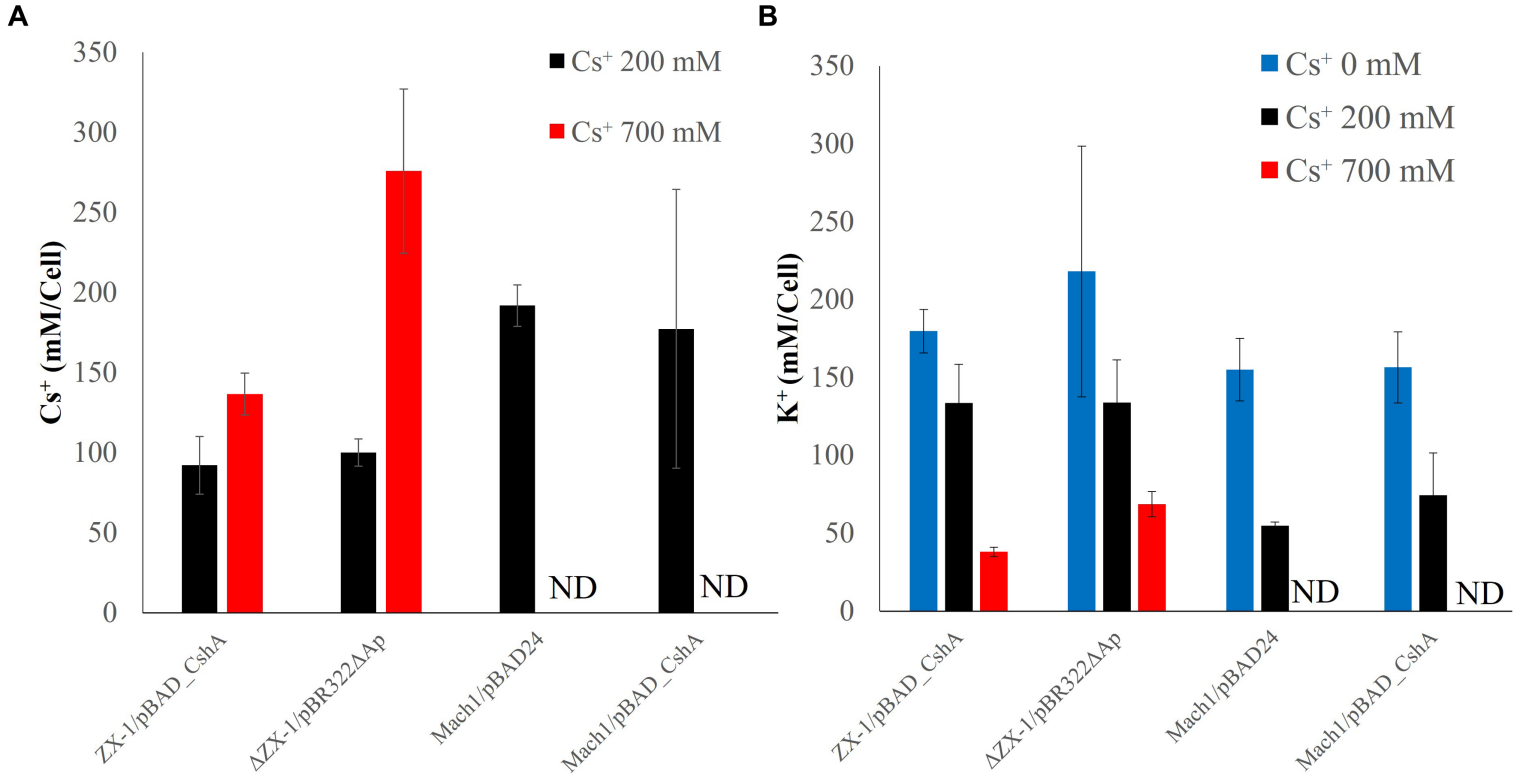
Intracellular Cs^+^ **(A)** and K^+^ **(B)** concentrations of transformed *E. coli* cultured at each Cs^+^ concentration. The Cs^+^ concentration conditions at which the cells were cultured are shown as 0 mM (blue), 200 mM (black), and 700 mM (red). The vertical axis indicates the Cs^+^ or K^+^ concentration, and the horizontal axis indicates the type of *E. coli* strain; Mach1/pBAD24 and Mach1/pBAD_CshA are labeled ND (not done) because they cannot grow in the presence of 700 mM Cs. Error bars also indicate the standard deviation of three independent experiments.

The Cs^+^/H^+^ antiport assay indicated that the strain ΔZX-1/pBR322ΔAp lacks CshA. Therefore, to elucidate why ΔZX-1/pBR322ΔAp can grow in high Cs^+^ concentrations, we analyzed the intracellular K^+^ and Cs^+^ concentrations in strains Mach1^™^/pBAD24, Mach1^™^/pBAD_CshA, ZX-1, and ΔZX-1/pBR322ΔAp in the presence of Cs^+^. Notably, strains ZX-1 and ΔZX-1/pBR322ΔAp maintained higher intracellular K^+^ concentrations compared to strains Mach1^™^/pBAD24 and Mach1^™^/pBAD_CshA when exposed to 200 mM CsCl (Figure 5B). In the strain Mach1^™^/pBAD24, which lacks a Cs^+^ resistance mechanism, the intracellular Cs^+^ concentration was nearly equivalent to the extracellular concentration at 200 mM Cs^+^ (Figure 5A). A similar observation was made in Mach1^™^/pBAD_CshA under the same conditions. Conversely, under 200 mM Cs^+^ conditions, the intracellular Cs^+^ concentration in strains ZX-1 and ΔZX-1/pBR322ΔAp was approximately 100 mM, about half that of the external Cs^+^ environment. Moreover, under 700 mM Cs^+^ conditions, the intracellular Cs^+^ concentration in ΔZX-1/pBR322ΔAp was about double that of ZX-1.

### 3.5 Whole-genome sequencing analysis of Mach1^™^ and ZX-1

Whole-genome sequence analysis of the strains Mach1^™^ and ZX-1 revealed four single nucleotide polymorphisms (SNPs) within regions encoding functional genes on the chromosomes, differing from the parent strain Mach1^™^. These SNPs did not involve *E. coli* transporter-related genes. Additionally, one of the four mutations were synonymous substitutions. Three nonsynonymous substitutions were identified, affecting genes encoding the ribosomal bS6 modification enzyme RimK, the phage lysis regulatory protein LysB, and the flagellar basal body rod protein FlgG. Details of each mutation site are summarized in Table 4.

**Table 4:** Summary of amino acid mutation sites, accession numbers, and coding sequences of ZX-1.

Explanation of protein function and amino acid mutation sites (Mach1^™^ → ZX-1)	Accession number and coding sequence
FlgG (Locus tag: NC_01730)	DRA017250
flagellar basal body rod protein	1,728,063–1,728,848
L12I (C34A)	
LysB (Locus tag: NC_02369)	DRA017250
phage lysis regulatory protein	2,374,895–2,375,323
L23V (C67G)	
RimK (Locus tag: NC_02398)	DRA017250
ribosomal bS6 modification enzyme	2,395,629–2,396,531
A160G (C479G)	

### 3.6 Analysis of ribosomes by sucrose density gradient ultracentrifugation

**Figure 6:**
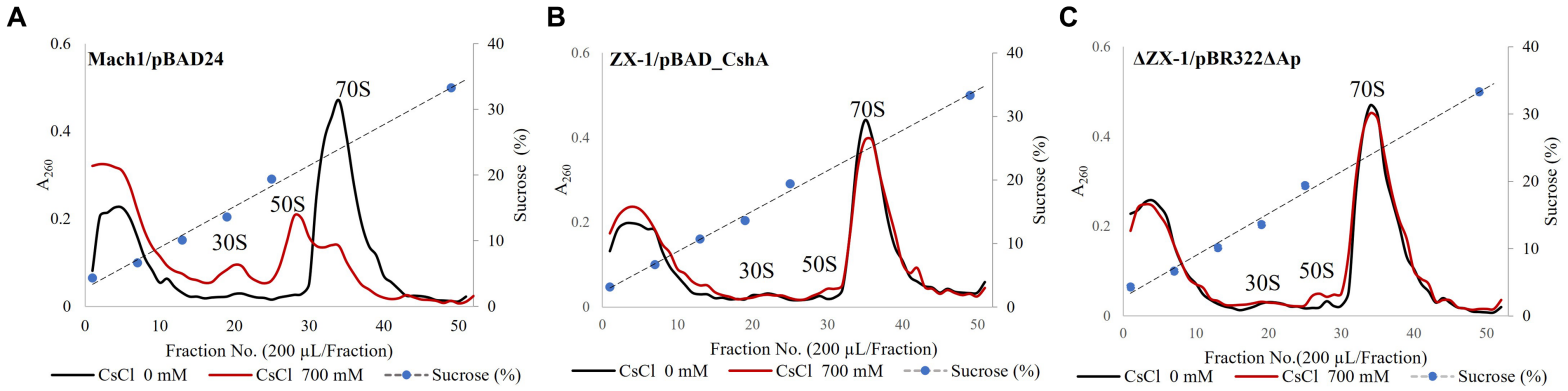
Analysis of ribosomes by sucrose density gradient ultracentrifugation. The effect of CsCl treatment of Strains Mach1^™^/pBAD24 **(A)**, ZX-1/pBAD_CshA **(B)**, and ΔZX-1/pBR322ΔAp **(C)** on the ribosomal complex was evaluated. Cs^+^ treatment was performed at 700 mM CsCl. Each crude cell extract was analyzed for ribosomes using sucrose density gradient ultracentrifugation. The first vertical axis indicates absorbance at A_260_. The second vertical axis indicates sucrose concentration. The abscissa shows the fraction number. Experiments were carried out three times for reproducibility.

The intracellular Cs^+^ concentration of the strain ΔZX-1/pBR322ΔAp, when cultured with 700 mM Cs^+^, was about twice that of the strain ZX-1 (Figure 5A). In addition, the intracellular K^+^ concentrations of the strains ZX-1 and ΔZX-1/pBR322ΔAp were like those of the strains Mach1TM/pBAD24 and Mach1TM/pBAD_CshA when 200 mM Cs^+^ was added (Figure 5B). Therefore, to assess whether high intracellular Cs^+^ or insufficient K^+^ affects the formation of ribosomal complexes in the strain ΔZX-1/pBR322ΔAp, we tested the fractionation of ribosomes at no Cs^+^ and 700 mM Cs^+^ addition by sucrose density gradient ultracentrifugation. Cs^+^-sensitive strain Mach1^™^/pBAD24, 70S ribosomes collapsed. Peaks were observed at 50S ribosomes, 30S ribosomes, and smaller sizes under the condition of 700 mM CsCl added (Figure 6A). On the other hand, in the case of the Cs^+^-resistant strain ZX-1, no effect on the ribosomal complex was observed with or without the addition of 700 mM CsCl (Figure 6B). No effect on the ribosomal complex was also observed in the strain ΔZX-1/pBR322ΔAp with or without the addition of 700 mM CsCl (Figure 6C).

## 4 Discussion

### 4.1 Isolation of strain ZX-1: improvement of Cs^+^ resistance in *Escherichia coli* without mutation of introduced plasmid

In our pursuit of increasing the substrate affinity of the CshA antiporter for Cs^+^, we were unsuccessful in introducing beneficial mutations into the *TS_cshA* gene. However, we obtained strain ZX-1 resistant to high concentrations of Cs^+^. The emergent *E. coli* strain ZX-1, which thrives in media with Cs^+^ concentrations as high as 400 mM, significantly advances our understanding of Cs^+^ resistance. Notably, the growth rate of ZX-1 was comparable to that of the Mach1^™^ in the absence of Cs^+^ (Figure 2A), indicating that the mutations did not compromise general cellular functions.

An intriguing result is the inability to replicate the Cs^+^ resistance observed in ZX-1 by reintroducing the pBAD_CshA plasmid into the parental Mach1^™^ strain. This suggests that the resistance phenotype of ZX-1 may stem from factors other than the plasmid-borne *TS_cshA*. This was further corroborated by the 100% homology between the plasmids pBAD_CshA from ZX-1 and pBAD_CshA from Mach1^™^ and the absence of detectable mutations within the plasmid sequence. Given that there are no known reports of Cs^+^-resistant *E. coli* strains to date, our findings suggest a potentially novel Cs^+^ resistance mechanism within ZX-1. This chromosomally encoded mechanism suggests potential new research directions in understanding the adaptability and resilience of *E. coli* to Cs^+^ stress. Additionally, examining the regulatory networks that assist ZX-1 in withstanding Cs^+^ stress could provide insights into bacterial survival strategies and possibly identify targets for engineering microbial strains with specialized cation resistance profiles.

### 4.2 Unveiling the cesium resistance mechanisms in *Escherichia coli* strains ZX-1 and ΔZX-1

The variance in Cs^+^ resistance observed among the *E. coli* strains in our study underscores the complex nature of Cs^+^ tolerance mechanisms. The growth inhibition of strain Mach1^™^/pBAD24 at a Cs^+^ concentration of 300 mM is consistent with previous observations of Cs^+^ sensitivity in *E. coli*, as reported by Ishida et al. (2023a) for *E. coli* W3110. However, the extraordinary resistance of strain ZX-1, which thrived at 800 mM Cs^+^, indicates its unique resistance capabilities, which merits further exploration.

Particularly intriguing is the growth of strain ΔZX-1/pBR322ΔAp at 700 mM Cs^+^, despite the removal of the pBAD_CshA plasmid, which is known to confer Cs^+^ resistance. This unanticipated finding strongly suggests the existence of an alternative Cs^+^ resistance mechanism intrinsic to the ΔZX-1 genome and not relying on the CshA antiporter.

The restoration of Cs^+^ resistance in ΔZX-1 upon reintroduction of pBAD_CshA, allowing growth at 800 mM CsCl, further indicates that the presence of CshA amplifies resistance. This amplification may result from the contribution of CshA to an already robust intrinsic resistance mechanism within the ΔZX-1 rather than CshA acting as the sole resistance factor. Our findings suggest the importance of identifying the genetic basis of innate Cs^+^ tolerance in ΔZX-1. This knowledge might offer useful insights into bacterial adaptation processes and potentially aid in the development of strains with specific resistance characteristics.

Future research could benefit from focusing on isolating and characterizing the genes responsible for the high Cs^+^ resistance observed in ZX-1 and ΔZX-1/pBR322ΔAp. Gaining an understanding of the functional dynamics between these genes and the CshA antiporter would be valuable, as it might uncover new strategies to enhance Cs^+^ resistance. Further exploration of the cellular mechanisms enabling *E. coli* to withstand high Cs^+^ concentrations without compromising growth would contribute to a more comprehensive understanding of Cs^+^ resistance.

### 4.3 Elucidating Cs^+^ resistance and antiport activity in *Escherichia coli*: the enhanced substrate affinity of CshA

The observed resistance of the *E. coli* strain ΔZX-1/pBR322ΔAp to high concentrations of Cs^+^ has led to reevaluating the mechanisms involved in Cs^+^ resistance. In the Cs^+^ resistance assay, this strain exhibited a level of resistance that was not anticipated, prompting further consideration regarding the potential completeness of the pBAD_CshA plasmid removal. Additional analysis of Cs^+^/H^+^ antiport activity and substrate affinity across various strains, including Mach1^™^/pBAD24, ZX-1, ΔZX-1/pBR322ΔAp, and ΔZX-1 reintroduced with pBAD_CshA, has provided us with more comprehensive insights.

The observed apparent *K*_m_ value of 28.7 mM (pH 8.5) for Strain ZX-1 indicates a significant increase in substrate affinity for Cs^+^, contrasting sharply with the previously reported affinity of 370 mM (pH 8.5) for TS_CshA. This 12.9-fold increase suggests a significant enhancement in Cs^+^-binding efficiency, which could be attributed to the genetic adaptations that have occurred in ZX-1.

The lack of antiport activity in strain ΔZX-1/pBR322ΔAp further corroborates the presence of an alternative Cs^+^ resistance mechanism. This finding aligns with our earlier hypothesis that ZX-1 possesses a Cs^+^ resistance mechanism distinct from that of the CshA antiporter.

Moreover, the retransformation of ΔZX-1/pBAD_CshA, resulting in an apparent *K*_m_ value of about 43.8 mM, indicates a reduced, yet still notable, substrate affinity compared to ZX-1. However, this affinity was sufficiently high to facilitate active Cs^+^/H^+^ antiport at concentrations lower than that of strain Mach1^™^/pBAD_CshA. This suggests that not only does ZX-1 exhibit a novel mechanism of Cs^+^ resistance, but it may also be capable of enhancing the functionality of the CshA antiporter.

These findings suggest that there might be yet-to-be-discovered genetic or epigenetic factors influencing the activity of the antiporter and overall cell resistance to Cs^+^. To comprehensively understand the mechanisms driving this enhanced resistance and antiport activity, future research should emphasize the genetic characterization of ZX-1 and ΔZX-1/pBR322ΔAp. Additionally, investigating potential regulatory pathways contributing to the increased efficiency of Cs^+^ transport could unveil new avenues for engineering microbial strains tailored for the environmental detoxification of cesium pollutants. The cell membrane lipids of Mach1^™^/pBAD24 and ZX-1 will be analyzed using gas chromatography to elucidate these factors. Furthermore, following Cs^+^ treatment, changes in protein profiles within each bacterial cell will be examined using two-dimensional electrophoresis, and alterations in gene expression will be assessed through RNA-Seq analysis.

### 4.4 Mechanisms of cesium-resistance in *Escherichia coli*: insights from intracellular ion dynamics

Our investigation into the Cs^+^/H^+^ antiport activity of *E. coli* strains revealed intricate details regarding their survival strategies in Cs^+^-rich environments. Notably, strain ΔZX-1/pBR322ΔAp, which lacks the CshA protein based on the Cs^+^/H^+^ antiport assay, presented an intriguing case of Cs^+^ resistance. This prompted us to examine the intracellular dynamics of K^+^ and Cs^+^ when the strains were exposed to Cs^+^.

Our measurements demonstrated a universal decrease in intracellular K^+^ concurrent with an increase in Cs^+^ across all strains. This observation is consistent with the hypothesis presented in the introduction that the K^+^ uptake system is competitively inhibited by Cs^+^, which is also supported by Rozov et al. (2019). Specifically, strains Mach1^™^/pBAD24 and Mach1^™^/pBAD_CshA exhibited lower intracellular K^+^ concentrations compared to strains ZX-1 and ΔZX-1/pBR322ΔAp, implicating this decrease in K^+^ as a potential factor in their growth inhibition.

At a Cs^+^ concentration of 200 mM, Mach1^™^ strains had intracellular Cs^+^ concentrations equivalent to their extracellular environment, suggesting a lack of Cs^+^ efflux capability, particularly in strain Mach1^™^/pBAD24, which lacks functional CshA. In contrast, strain Mach1^™^/pBAD_CshA, despite possessing CshA, may be ineffective due to the environmental Cs^+^ concentration being below the apparent *K*_m_ value of the antiporter.

Conversely, ZX-1 and ΔZX-1/pBR322ΔAp maintained intracellular Cs^+^ concentrations at approximately half the external levels under the same conditions. This remarkable capability implies the presence of an alternative mechanism in ΔZX-1/pBR322ΔAp that helps maintain lower intracellular Cs^+^ concentrations. Even more intriguingly, at 700 mM Cs^+^, strain ΔZX-1/pBR322ΔAp showed an intracellular Cs^+^ concentration about twice that of ZX-1, yet both strains were capable of growth, as evidenced in the Cs^+^ resistance assays (Figure 5A). This suggests that both strains possess mechanisms that permit their survival at high intracellular Cs^+^ or low K^+^ concentrations.

The precise nature of these mechanisms, however, is not yet exact. Nonetheless, these findings suggest that Cs^+^ resistance in *E. coli* could involve more complex physiological adaptations beyond the roles of known antiporters. Future research may benefit from identifying these mechanisms and understanding how they confer resistance at a molecular level. Such knowledge might offer new possibilities for bioengineering strains with enhanced resistance to heavy metal stress, which could be helpful in the bioremediation of environments contaminated with toxic metals.

### 4.5 Genomic insights into Cs^+^ resistance mechanisms in *Escherichia coli* strain ZX-1

Whole-genome analysis of the highly Cs^+^-resistant *E. coli* strain ZX-1 revealed several SNPs distinct from the parent strain Mach1^™^. Notably, no transporter-related genes previously hypothesized to be involved in potential Cs^+^ resistance mechanisms were identified. However, three significant mutations were detected in genes encoding the ribosomal bS6 modification enzyme RimK, the phage lysis regulatory protein LysB and the flagellar base component protein FlgG. These mutations are potential candidates that alter protein function.

The ribosomal bS6 modification enzyme RimK, a member of the ATP-dependent carboxylate amine/thiol ligase superfamily (Galperin and Koonin, 1997), is thought to modify ribosomal protein bS6 in an ATP-dependent manner, though its precise role remains unclear (Thompson et al., 2023). Phage lysis regulatory proteins belonging to the LysB family also have unknown functions (Pimentel, 2014). FlgG protein is a constituent protein of the flagellar motor (Nakamura and Minamino, 2019), and it is currently unknown whether it is associated with the physiological phenotype of improved Cs^+^ tolerance in ZX-1.

Our findings are particularly significant considering our prior research, which indicated that ribosomes are destabilized in the presence of Cs^+^ but can be stabilized by magnesium ions (Ishida et al., 2023b). In addition, K^+^ has been reported to play a role in ribosome stabilization (Nierhaus, 2014; Rozov et al., 2019). These results suggest that the identified ribosomal mutations may contribute to enhanced Cs^+^ resistance in *E. coli*.

Furthermore, re-transforming strain ΔZX-1/pBR322ΔAp with pBAD_CshA increased TS_CshA substrate affinity for Cs^+^. This is reminiscent of the YfkE Ca^2+^/H^+^ antiporter, which transports calcium (Ca^2+^) in two modes. One is a low-flux H^+^/Ca^2+^ exchange mode, and the other is a high-flux cotransport mode of Ca^2+^ and phosphate ions. This co-transport mode enabled efficient excretion of Ca^2+^ and revealed a novel co-transport mechanism within the CAX family (Niu et al., 2023). The role of small subunits, such as the 29 amino acid residue KdpF, in the *E. coli* K^+^ uptake system (Gassel et al., 1999) and the influence of phospholipids, such as cardiolipin, on membrane protein activity (Arias-Cartin et al., 2012) further support this hypothesis. Given that cardiolipin concentration varies significantly among organisms (Lin and Weibel, 2016), the increased affinity of TS_CshA for ZX-1 may be influenced by proteins or membrane lipids specific to this strain. Future studies should explore changes in cardiolipin expression and localization in the membrane lipid composition of *E. coli* Mach1^™^ and strain ΔZX-1/pBR322ΔAp to understand these mechanisms better.

### 4.6 Robustness of ribosomes may contribute to Cs^+^ resistance

This experiment investigated the impact on ribosomes when *E. coli* transformants were exposed to Cs^+^. In previous reports, the ribosome complex of Cs^+^-sensitive mutants of *Microbacterium* sp. TS-1, a Cs^+^ resistant bacterium, exhibited 70S ribosomal disintegration in the presence of 200 mM and 400 mM Cs^+^, which was not observed in the wild-type. When *E. coli* strain Mach1^™^/pBAD24 was treated with 700 mM Cs^+^, the ribosomal breakdown occurred, with peaks observed at the 50S, 30S, and smaller sizes (Figure 6A). In the strain ZX-1 possessing the Cs^+^ efflux mechanism CshA, the ribosome complex remained unaffected regardless of Cs^+^ treatment (Figure 6B). Remarkably, even in the strain ΔZX-1/pBR322ΔAp, where CshA was removed, the robustness of ribosome complexes unaffected by Cs^+^ treatment was confirmed (Figure 6C). The intracellular K^+^ concentration of strain ΔZX-1/pBR322ΔAp cultured in 700 mM CsCl was nearly equivalent to that of strains Mach1^™^/pBAD24 and Mach1^™^/pBAD_CshA cultured in the presence of 200 mM CsCl, and the intracellular Cs^+^ concentration was approximately twice that of the strain ZX-1 (Figure 5B). Nevertheless, the ribosome complex was minimally affected. As previously described, ribosomes become unstable in the presence of Cs^+^. Therefore, it is suggested that ribosomes may have overcome Cs^+^-induced destabilization. Notably, despite our previous report’s minimal impact on ribosome complexes due to Cs^+^ treatment, the *Bacillus subtilis* did not exhibit Cs^+^ resistance (Ishida et al., 2023a). This suggests that the destabilization of ribosomes alone may not be the direct cause of cell death in bacteria exposed to Cs^+^. The rapid decrease in K^+^ accompanying the sudden increase in Cs^+^ could inhibit vital cellular activities due to K^+^ deficiency. For example, in bacteria, K^+^ is known to maintain and regulate responses to osmotic stress, pH stress, membrane potential, and the expression and activity of genes and enzymes (Ballal et al., 2007; Beagle and Lockless, 2021; Stautz et al., 2021). Therefore, in Cs^+^-resistant *E. coli* strain ZX-1, in addition to robust ribosomes, there may be a complex Cs^+^ resistance mechanism to adapt to K^+^ deficiency.

## 5 Conclusion

This study reveals that the highly Cs^+^-resistant strain ZX-1, which emerged unexpectedly from *E. coli* Mach1^™^ transformed with the pBAD_CshA plasmid, possesses a unique Cs^+^ resistance mechanism distinct from TS_CshA. In addition, the ZX-1 may exhibit increased substrate affinity for TS_CshA due to factors unrelated to the CshA mutation. It has been suggested that the robustness of the 70S ribosome complex of the ΔZX-1/pBR322ΔAp contributes to its Cs^+^ resistance, even under high intracellular Cs^+^ levels. Further, this research indicates that mutations in the genomic DNA of *E. coli* enhance Cs^+^ resistance. Genome analysis of ZX-1 has identified three mutations, one of which is associated with ribosome genes. Previous studies have shown that ribosomal robustness affects Cs^+^ resistance. These mutations might also lower apparent *K*_m_ values by altering accessory protein expression and changing the composition of membrane lipids. Future research will determine if introducing these mutated genes into the parent Mach1^™^ enhances Cs^+^ resistance. Moreover, changes in membrane lipid composition will be examined. Identifying genes related to Cs^+^ resistance could elucidate the mechanism of Cs^+^ resistance in *E. coli* and other organisms. This study also lays the groundwork for applied research using the Cs^+^ resistance mechanism. For instance, employing inverted membrane vesicles expressing TS_CshA could lead to the development of technologies for efficiently recovering radioactive Cs^+^ from contaminated water.

## Data availability statement

The datasets presented in this study can be found in online repositories. The names of the repository/repositories and accession number(s) can be found at: https://www.ddbj.nig.ac.jp/, DRA017249; https://www.ddbj.nig.ac.jp/, DRA017250.

## Author contributions

DK: Data curation, Formal analysis, Methodology, Writing – original draft, Writing – review & editing. ST: Data curation, Formal analysis, Validation, Writing – review & editing. AK: Validation, Visualization, Writing - review & editing. XZ: Data curation, Formal analysis, Validation, Writing – review & editing. MI: Data curation, Formal analysis, Funding acquisition, Investigation, Project administration, Supervision, Validation, Writing – original draft, Writing – review & editing.
