# Supplementary material for: Isolation and Cs+ resistance mechanism of Escherichia coli strain ZX-1
Source: Front Microbiol. 2024 Jan 18;14:1340033. doi: 10.3389/fmicb.2023.1340033 (PMC10831881; doi:10.3389/fmicb.2023.1340033)
Supplement: Supplementary file 1 [file Data_Sheet_1.PDF]

Additional file 1

Isolation and Cs^+ Resistance Mechanism of *Escherichia coli* Strain ZX-1

Daiki Kojima1, Shun Tanaka2, Xiong Zhiyu2 and Masahiro Ito1,2,3,4*
1Graduate School of Life Sciences, Toyo University, Oura-gun, Gunma 374-0193 Japan
2Faculty of Life Sciences, Toyo University, Oura-gun, Gunma 374-0193 Japan
3Bio-Nano Electronics Research Center, Toyo University, Kawagoe, Saitama 350-8585 Japan
4Bio-resilience research project (BRRP), Toyo University, Oura-gun, Gunma 374-0193 Japan

Supplementary Table S1. Number of mutated bases and number of non-synonymous substituted amino acids in the *cshA* gene PCR product obtained from three independent Error Prone PCRs

Colony number	Number of mutated bases/*cshA* gene (1560 bp)	Number of non-synonymous substituted amino acids /CshA (520aa)
No.1-1	0	0
No. 1-2	0	0
No. 1-3	0	0
No. 1-4	0	0
No. 1-5	0	0
Ave. (No. 1)	0	0
No. 2-1	9	4
No.2-2	10	6
No.2-3	10	5
No.2-4	15	6
No.2-5	16	9
Ave. (No. 2)	12.0	6.0
No. 3-1	16	5
No.3-2	10	5
No. 3-3	22	8
No. 3-4	14	6
No. 3-5	18	6
Ave. (No. 3).	16.0	6.0

According to the instruction manual of the Diversify PCR Random Mutagenesis Kit (TAKARA BIO Inc., Kyoto, Japan), experiments were conducted under conditions that introduced 6 to 8 bases per 1000 bp mutations. Plasmid DNA was prepared from colonies obtained in three independent experiments, and the cshA gene sequence was DNA sequenced using the Sanger method.

pBAD_CshA_ Length: 6087

Score:11241 bits(6087), Expect:0.0,
Identities:6087/6087(100%), Gaps:0/6087(0%), Strand: Plus/Plus

```
pBAD_CshA  1     TCTTCCGCTTCCTCGCTCACTGACTCGCTGCGCTCGGTCGTTCGGCTGCGGCGAGCGGTA  60
                 ||||||||||||||||||||||||||||||||||||||||||||||||||||||||||||
pZX_CshA   1     TCTTCCGCTTCCTCGCTCACTGACTCGCTGCGCTCGGTCGTTCGGCTGCGGCGAGCGGTA  60

pBAD_CshA  61    TCAGCTCACTCAAAGGCGGTAATACGGTTATCCACAGAATCAGGGGATAACGCAGGAAAG  120
                 ||||||||||||||||||||||||||||||||||||||||||||||||||||||||||||
pZX_CshA   61    TCAGCTCACTCAAAGGCGGTAATACGGTTATCCACAGAATCAGGGGATAACGCAGGAAAG  120

pBAD_CshA  121   AACATGTGAGCAAAAGGCCAGCAAAAGGCCAGGAACCGTAAAAAGGCCGCGTTGCTGGCG  180
                 ||||||||||||||||||||||||||||||||||||||||||||||||||||||||||||
pZX_CshA   121   AACATGTGAGCAAAAGGCCAGCAAAAGGCCAGGAACCGTAAAAAGGCCGCGTTGCTGGCG  180

pBAD_CshA  181   TTTTTCCATAGGCTCCGCCCCCCTGACGAGCATCACAAAAATCGACGCTCAAGTCAGAGG  240
                 ||||||||||||||||||||||||||||||||||||||||||||||||||||||||||||
pZX_CshA   181   TTTTTCCATAGGCTCCGCCCCCCTGACGAGCATCACAAAAATCGACGCTCAAGTCAGAGG  240

pBAD_CshA  241   TGGCGAAACCCGACAGGACTATAAAGATACCAGGCGTTTCCCCCTGGAAGCTCCCTCGTG  300
                 ||||||||||||||||||||||||||||||||||||||||||||||||||||||||||||
pZX_CshA   241   TGGCGAAACCCGACAGGACTATAAAGATACCAGGCGTTTCCCCCTGGAAGCTCCCTCGTG  300

pBAD_CshA  301   CGCTCTCCTGTTCCGACCCTGCCGCTTACCGGATACCTGTCCGCCTTTCTCCCTTCGGGA  360
                 ||||||||||||||||||||||||||||||||||||||||||||||||||||||||||||
pZX_CshA   301   CGCTCTCCTGTTCCGACCCTGCCGCTTACCGGATACCTGTCCGCCTTTCTCCCTTCGGGA  360

pBAD_CshA  361   AGCGTGGCGCTTTCTCATAGCTCACGCTGTAGGTATCTCAGTTCGGTGTAGGTCGTTCGC  420
                 ||||||||||||||||||||||||||||||||||||||||||||||||||||||||||||
pZX_CshA   361   AGCGTGGCGCTTTCTCATAGCTCACGCTGTAGGTATCTCAGTTCGGTGTAGGTCGTTCGC  420

pBAD_CshA  421   TCCAAGCTGGGCTGTGTGCACGAACCCCCCGTTCAGCCCGACCGCTGCGCCTTATCCGGT  480
                 ||||||||||||||||||||||||||||||||||||||||||||||||||||||||||||
pZX_CshA   421   TCCAAGCTGGGCTGTGTGCACGAACCCCCCGTTCAGCCCGACCGCTGCGCCTTATCCGGT  480

pBAD_CshA  481   AACTATCGTCTTGAGTCCAACCCGGTAAGACACGACTTATCGCCACTGGCAGCAGCCACT  540
                 ||||||||||||||||||||||||||||||||||||||||||||||||||||||||||||
pZX_CshA   481   AACTATCGTCTTGAGTCCAACCCGGTAAGACACGACTTATCGCCACTGGCAGCAGCCACT  540

pBAD_CshA  541   GGTAACAGGATTAGCAGAGCGAGGTATGTAGGCGGTGCTACAGAGTTCTTGAAGTGGTGG  600
                 ||||||||||||||||||||||||||||||||||||||||||||||||||||||||||||
pZX_CshA   541   GGTAACAGGATTAGCAGAGCGAGGTATGTAGGCGGTGCTACAGAGTTCTTGAAGTGGTGG  600

pBAD_CshA  601   CCTAACTACGGCTACACTAGAAGGACAGTATTTGGTATCTGCGCTCTGCTGAAGCCAGTT  660
                 ||||||||||||||||||||||||||||||||||||||||||||||||||||||||||||
pZX_CshA   601   CCTAACTACGGCTACACTAGAAGGACAGTATTTGGTATCTGCGCTCTGCTGAAGCCAGTT  660

pBAD_CshA  661   ACCTTCGGAAAAAGAGTTGGTAGCTCTTGATCCGGCAAACAAACCACCGCTGGTAGCGGT  720
                 ||||||||||||||||||||||||||||||||||||||||||||||||||||||||||||
pZX_CshA   661   ACCTTCGGAAAAAGAGTTGGTAGCTCTTGATCCGGCAAACAAACCACCGCTGGTAGCGGT  720

pBAD_CshA  721   GGTTTTTTTGTTTGCAAGCAGCAGATTACGCGCAGAAAAAAAGGATCTCAAGAAGATCCT  780
                 ||||||||||||||||||||||||||||||||||||||||||||||||||||||||||||
pZX_CshA   721   GGTTTTTTTGTTTGCAAGCAGCAGATTACGCGCAGAAAAAAAGGATCTCAAGAAGATCCT  780
```

```
pBAD_CshA  781   TTGATCTTTTCTACGGGGTCTGACGCTCAGTGGAACGAAAACTCACGTTAAGGGATTTTG  840
                 ||||||||||||||||||||||||||||||||||||||||||||||||||||||||||||
pZX_CshA   781   TTGATCTTTTCTACGGGGTCTGACGCTCAGTGGAACGAAAACTCACGTTAAGGGATTTTG  840

pBAD_CshA  841   GTCATGAGATTATCAAAAAGGATCTTCACCTAGATCCTTTTAAATTGTAAACGTTAATAT  900
                 ||||||||||||||||||||||||||||||||||||||||||||||||||||||||||||
pZX_CshA   841   GTCATGAGATTATCAAAAAGGATCTTCACCTAGATCCTTTTAAATTGTAAACGTTAATAT  900

pBAD_CshA  901   TTTGTTAAAATTCGCGTTAAATTTTTGTTAAATCAGCTCATTTTTTAACCAATAGGCCGA  960
                 ||||||||||||||||||||||||||||||||||||||||||||||||||||||||||||
pZX_CshA   901   TTTGTTAAAATTCGCGTTAAATTTTTGTTAAATCAGCTCATTTTTTAACCAATAGGCCGA  960

pBAD_CshA  961   AATCGGCAAAATCCCTTATAAATCAAAAGAATAGCCCGAGATAGGGTTGAGTGTTGTTCC  1020
                 ||||||||||||||||||||||||||||||||||||||||||||||||||||||||||||
pZX_CshA   961   AATCGGCAAAATCCCTTATAAATCAAAAGAATAGCCCGAGATAGGGTTGAGTGTTGTTCC  1020

pBAD_CshA  1021  AGTTTGGAACAAGAGTCCACTATTAAAGAACGTGGACTCCAACGTCAAAGGGCGAAAAAC  1080
                 ||||||||||||||||||||||||||||||||||||||||||||||||||||||||||||
pZX_CshA   1021  AGTTTGGAACAAGAGTCCACTATTAAAGAACGTGGACTCCAACGTCAAAGGGCGAAAAAC  1080

pBAD_CshA  1081  CGTCTATCAGGGCGATGGCCCACTACGTGAACCATCACCCAAATCAAGTTTTTTGGGGTC  1140
                 ||||||||||||||||||||||||||||||||||||||||||||||||||||||||||||
pZX_CshA   1081  CGTCTATCAGGGCGATGGCCCACTACGTGAACCATCACCCAAATCAAGTTTTTTGGGGTC  1140

pBAD_CshA  1141  GAGGTGCCGTAAAGCACTAAATCGGAACCCTAAAGGGAGCCCCCGATTTAGAGCTTGACG  1200
                 ||||||||||||||||||||||||||||||||||||||||||||||||||||||||||||
pZX_CshA   1141  GAGGTGCCGTAAAGCACTAAATCGGAACCCTAAAGGGAGCCCCCGATTTAGAGCTTGACG  1200

pBAD_CshA  1201  GGGAAAGCCGGCGAACGTGGCGAGAAAGGAAGGGAAGAAAGCGAAAGGAGCGGGCGCTAG  1260
                 ||||||||||||||||||||||||||||||||||||||||||||||||||||||||||||
pZX_CshA   1201  GGGAAAGCCGGCGAACGTGGCGAGAAAGGAAGGGAAGAAAGCGAAAGGAGCGGGCGCTAG  1260

pBAD_CshA  1261  GGCGCTGGCAAGTGTAGCGGTCACGCTGCGCGTAACCACCACACCCGCCGCGCTTAATGC  1320
                 ||||||||||||||||||||||||||||||||||||||||||||||||||||||||||||
pZX_CshA   1261  GGCGCTGGCAAGTGTAGCGGTCACGCTGCGCGTAACCACCACACCCGCCGCGCTTAATGC  1320

pBAD_CshA  1321  GCCGCTACAGGGCGCGTAAATCAATCTAAAGTATATATGAGTAAACTTGGTCTGACAGTT  1380
                 ||||||||||||||||||||||||||||||||||||||||||||||||||||||||||||
pZX_CshA   1321  GCCGCTACAGGGCGCGTAAATCAATCTAAAGTATATATGAGTAAACTTGGTCTGACAGTT  1380

pBAD_CshA  1381  ACCAATGCTTAATCAGTGAGGCACCTATCTCAGCGATCTGTCTATTTCGTTCATCCATAG  1440
                 ||||||||||||||||||||||||||||||||||||||||||||||||||||||||||||
pZX_CshA   1381  ACCAATGCTTAATCAGTGAGGCACCTATCTCAGCGATCTGTCTATTTCGTTCATCCATAG  1440

pBAD_CshA  1441  TTGCCTGACTCCCCGTCGTGTAGATAACTACGATACGGGAGGGCTTACCATCTGGCCCCA  1500
                 ||||||||||||||||||||||||||||||||||||||||||||||||||||||||||||
pZX_CshA   1441  TTGCCTGACTCCCCGTCGTGTAGATAACTACGATACGGGAGGGCTTACCATCTGGCCCCA  1500

pBAD_CshA  1501  GTGCTGCAATGATACCGCGAGACCCACGCTCACCGGCTCCAGATTTATCAGCAATAAACC  1560
                 ||||||||||||||||||||||||||||||||||||||||||||||||||||||||||||
pZX_CshA   1501  GTGCTGCAATGATACCGCGAGACCCACGCTCACCGGCTCCAGATTTATCAGCAATAAACC  1560

pBAD_CshA  1561  AGCCAGCCGGAAGGGCCGAGCGCAGAAGTGGTCCTGCAACTTTATCCGCCTCCATCCAGT  1620
                 ||||||||||||||||||||||||||||||||||||||||||||||||||||||||||||
pZX_CshA   1561  AGCCAGCCGGAAGGGCCGAGCGCAGAAGTGGTCCTGCAACTTTATCCGCCTCCATCCAGT  1620
```

```
pBAD_CshA  1621  CTATTAATTGTTGCCGGGAAGCTAGAGTAAGTAGTTCGCCAGTTAATAGTTTGCGCAACG  1680
                 ||||||||||||||||||||||||||||||||||||||||||||||||||||||||||||
pZX_CshA   1621  CTATTAATTGTTGCCGGGAAGCTAGAGTAAGTAGTTCGCCAGTTAATAGTTTGCGCAACG  1680

pBAD_CshA  1681  TTGTTGCCATTGCTACAGGCATCGTGGTGTCACGCTCGTCGTTTGGTATGGCTTCATTCA  1740
                 ||||||||||||||||||||||||||||||||||||||||||||||||||||||||||||
pZX_CshA   1681  TTGTTGCCATTGCTACAGGCATCGTGGTGTCACGCTCGTCGTTTGGTATGGCTTCATTCA  1740

pBAD_CshA  1741  GCTCCGGTTCCCAACGATCAAGGCGAGTTACATGATCCCCCATGTTGTGCAAAAAAGCGG  1800
                 ||||||||||||||||||||||||||||||||||||||||||||||||||||||||||||
pZX_CshA   1741  GCTCCGGTTCCCAACGATCAAGGCGAGTTACATGATCCCCCATGTTGTGCAAAAAAGCGG  1800

pBAD_CshA  1801  TTAGCTCCTTCGGTCCTCCGATCGTTGTCAGAAGTAAGTTGGCCGCAGTGTTATCACTCA  1860
                 ||||||||||||||||||||||||||||||||||||||||||||||||||||||||||||
pZX_CshA   1801  TTAGCTCCTTCGGTCCTCCGATCGTTGTCAGAAGTAAGTTGGCCGCAGTGTTATCACTCA  1860

pBAD_CshA  1861  TGGTTATGGCAGCACTGCATAATTCTCTTACTGTCATGCCATCCGTAAGATGCTTTTCTG  1920
                 ||||||||||||||||||||||||||||||||||||||||||||||||||||||||||||
pZX_CshA   1861  TGGTTATGGCAGCACTGCATAATTCTCTTACTGTCATGCCATCCGTAAGATGCTTTTCTG  1920

pBAD_CshA  1921  TGACTGGTGAGTACTCAACCAAGTCATTCTGAGAATAGTGTATGCGGCGACCGAGTTGCT  1980
                 ||||||||||||||||||||||||||||||||||||||||||||||||||||||||||||
pZX_CshA   1921  TGACTGGTGAGTACTCAACCAAGTCATTCTGAGAATAGTGTATGCGGCGACCGAGTTGCT  1980

pBAD_CshA  1981  CTTGCCCGGCGTCAACACGGGATAATACCGCGCCACATAGCAGAACTTTAAAAGTGCTCA  2040
                 ||||||||||||||||||||||||||||||||||||||||||||||||||||||||||||
pZX_CshA   1981  CTTGCCCGGCGTCAACACGGGATAATACCGCGCCACATAGCAGAACTTTAAAAGTGCTCA  2040

pBAD_CshA  2041  TCATTGGAAAACGTTCTTCGGGGCGAAAACTCTCAAGGATCTTACCGCTGTTGAGATCCA  2100
                 ||||||||||||||||||||||||||||||||||||||||||||||||||||||||||||
pZX_CshA   2041  TCATTGGAAAACGTTCTTCGGGGCGAAAACTCTCAAGGATCTTACCGCTGTTGAGATCCA  2100

pBAD_CshA  2101  GTTCGATGTAACCCACTCGTGCACCCAACTGATCTTCAGCATCTTTTACTTTCACCAGCG  2160
                 ||||||||||||||||||||||||||||||||||||||||||||||||||||||||||||
pZX_CshA   2101  GTTCGATGTAACCCACTCGTGCACCCAACTGATCTTCAGCATCTTTTACTTTCACCAGCG  2160

pBAD_CshA  2161  TTTCTGGGTGAGCAAAAACAGGAAGGCAAAATGCCGCAAAAAAGGGAATAAGGGCGACAC  2220
                 ||||||||||||||||||||||||||||||||||||||||||||||||||||||||||||
pZX_CshA   2161  TTTCTGGGTGAGCAAAAACAGGAAGGCAAAATGCCGCAAAAAAGGGAATAAGGGCGACAC  2220

pBAD_CshA  2221  GGAAATGTTGAATACTCATACTCTTCCTTTTTCAATATTATTGAAGCATTTATCAGGGTT  2280
                 ||||||||||||||||||||||||||||||||||||||||||||||||||||||||||||
pZX_CshA   2221  GGAAATGTTGAATACTCATACTCTTCCTTTTTCAATATTATTGAAGCATTTATCAGGGTT  2280

pBAD_CshA  2281  ATTGTCTCATGAGCGGATACATATTTGAATGTATTTAGAAAAATAAACAAAAGAGTTTGT  2340
                 ||||||||||||||||||||||||||||||||||||||||||||||||||||||||||||
pZX_CshA   2281  ATTGTCTCATGAGCGGATACATATTTGAATGTATTTAGAAAAATAAACAAAAGAGTTTGT  2340

pBAD_CshA  2341  AGAAACGCAAAAAGGCCATCCGTCAGGATGGCCTTCTGCTTAATTTGATGCCTGGCAGTT  2400
                 ||||||||||||||||||||||||||||||||||||||||||||||||||||||||||||
pZX_CshA   2341  AGAAACGCAAAAAGGCCATCCGTCAGGATGGCCTTCTGCTTAATTTGATGCCTGGCAGTT  2400

pBAD_CshA  2401  TATGGCGGGCGTCCTGCCCGCCACCCTCCGGGCCGTTGCTTCGCAACGTTCAAATCCGCT  2460
                 ||||||||||||||||||||||||||||||||||||||||||||||||||||||||||||
pZX_CshA   2401  TATGGCGGGCGTCCTGCCCGCCACCCTCCGGGCCGTTGCTTCGCAACGTTCAAATCCGCT  2460
```

```
pBAD_CshA  2461  CCCGGCGGATTTGTCCTACTCAGGAGAGCGTTCACCGACAAACAACAGATAAAACGAAAG  2520
                 ||||||||||||||||||||||||||||||||||||||||||||||||||||||||||||
pZX_CshA   2461  CCCGGCGGATTTGTCCTACTCAGGAGAGCGTTCACCGACAAACAACAGATAAAACGAAAG  2520

pBAD_CshA  2521  GCCCAGTCTTTCGACTGAGCCTTTCGTTTTATTTGATGCCTGGCAGTTCCCTACTCTCGC  2580
                 ||||||||||||||||||||||||||||||||||||||||||||||||||||||||||||
pZX_CshA   2521  GCCCAGTCTTTCGACTGAGCCTTTCGTTTTATTTGATGCCTGGCAGTTCCCTACTCTCGC  2580

pBAD_CshA  2581  ATGGGGAGACCCCACACTACCATCGGCGCTACGGCGTTTCACTTCTGAGTTCGGCATGGG  2640
                 ||||||||||||||||||||||||||||||||||||||||||||||||||||||||||||
pZX_CshA   2581  ATGGGGAGACCCCACACTACCATCGGCGCTACGGCGTTTCACTTCTGAGTTCGGCATGGG  2640

pBAD_CshA  2641  GTCAGGTGGGACCACCGCGCTACTGCCGCCAGGCAAATTCTGTTTTATCAGACCGCTTCT  2700
                 ||||||||||||||||||||||||||||||||||||||||||||||||||||||||||||
pZX_CshA   2641  GTCAGGTGGGACCACCGCGCTACTGCCGCCAGGCAAATTCTGTTTTATCAGACCGCTTCT  2700

pBAD_CshA  2701  GCGTTCTGATTTAATCTGTATCAGGCTGAAAATCTTCTCTCATCCGCCAAAACAGCCAAG  2760
                 ||||||||||||||||||||||||||||||||||||||||||||||||||||||||||||
pZX_CshA   2701  GCGTTCTGATTTAATCTGTATCAGGCTGAAAATCTTCTCTCATCCGCCAAAACAGCCAAG  2760

pBAD_CshA  2761  CTTGCATGCCTGCAGGTCGACTCTAGATCAATGGTGATGGTGGTGATGTTCCGCCGGGCG  2820
                 ||||||||||||||||||||||||||||||||||||||||||||||||||||||||||||
pZX_CshA   2761  CTTGCATGCCTGCAGGTCGACTCTAGATCAATGGTGATGGTGGTGATGTTCCGCCGGGCG  2820

pBAD_CshA  2821  ACTATGCGCGCCTTTAAGGGTGGTTGCGGCAATGATGCCAGCGCAGACTACCAACACGGC  2880
                 ||||||||||||||||||||||||||||||||||||||||||||||||||||||||||||
pZX_CshA   2821  ACTATGCGCGCCTTTAAGGGTGGTTGCGGCAATGATGCCAGCGCAGACTACCAACACGGC  2880

pBAD_CshA  2881  ACCAATCGTCGCGGTTACACCTACTCCTGAATCGAAAGCATGCGCTGCTGCATCCAGCAG  2940
                 ||||||||||||||||||||||||||||||||||||||||||||||||||||||||||||
pZX_CshA   2881  ACCAATCGTCGCGGTTACACCTACTCCTGAATCGAAAGCATGCGCTGCTGCATCCAGCAG  2940

pBAD_CshA  2941  CTGGCGACCAACGGAATCCGGTAAGTCCTGAGCGACATTAACAGCGCCTGCTAATGTCTC  3000
                 ||||||||||||||||||||||||||||||||||||||||||||||||||||||||||||
pZX_CshA   2941  CTGGCGACCAACGGAATCCGGTAAGTCCTGAGCGACATTAACAGCGCCTGCTAATGTCTC  3000

pBAD_CshA  3001  ACGAGCAGCGTCAGCTGCTTCTGCCGGAACACCTTCCGGAACGGCCATCCCCAGGCGATA  3060
                 ||||||||||||||||||||||||||||||||||||||||||||||||||||||||||||
pZX_CshA   3001  ACGAGCAGCGTCAGCTGCTTCTGCCGGAACACCTTCCGGAACGGCCATCCCCAGGCGATA  3060

pBAD_CshA  3061  CATGGCGGTAAGGATACCACCAAGTACCGCTGTGCCCAGTACCGCTCCCACCTCATAGGC  3120
                 ||||||||||||||||||||||||||||||||||||||||||||||||||||||||||||
pZX_CshA   3061  CATGGCGGTAAGGATACCACCAAGTACCGCTGTGCCCAGTACCGCTCCCACCTCATAGGC  3120

pBAD_CshA  3121  CGTTTCCGATACGGCACTCGCTGCGCCTGCTTTCGCCGGTGGGGCACTAGCCAATACCAG  3180
                 ||||||||||||||||||||||||||||||||||||||||||||||||||||||||||||
pZX_CshA   3121  CGTTTCCGATACGGCACTCGCTGCGCCTGCTTTCGCCGGTGGGGCACTAGCCAATACCAG  3180

pBAD_CshA  3181  TTCGTTGCTAACGGTTTCGGCGGCCCCAATACCCATCCCCAACAGCGCAAATGCGAGCAC  3240
                 ||||||||||||||||||||||||||||||||||||||||||||||||||||||||||||
pZX_CshA   3181  TTCGTTGCTAACGGTTTCGGCGGCCCCAATACCCATCCCCAACAGCGCAAATGCGAGCAC  3240

pBAD_CshA  3241  CAGTTCGATCATGCTGTCAGGTTCCGTCAGCAGAGCAATCATTACGTAGCCCGCTGCACT  3300
                 ||||||||||||||||||||||||||||||||||||||||||||||||||||||||||||
pZX_CshA   3241  CAGTTCGATCATGCTGTCAGGTTCCGTCAGCAGAGCAATCATTACGTAGCCCGCTGCACT  3300
```

```
pBAD_CshA  3301  CAGAGTCAGGGCAGCAGGGACCACGATGCGTGGGCTGACACGGCGGCTGATCGGAACCAC  3360
                 ||||||||||||||||||||||||||||||||||||||||||||||||||||||||||||
pZX_CshA   3301  CAGAGTCAGGGCAGCAGGGACCACGATGCGTGGGCTGACACGGCGGCTGATCGGAACCAC  3360

pBAD_CshA  3361  ACCCAATCCAGCAACAATCATCATGGCCAGGCCCGGAACTAATGCAAGACCCGCCTGAAG  3420
                 ||||||||||||||||||||||||||||||||||||||||||||||||||||||||||||
pZX_CshA   3361  ACCCAATCCAGCAACAATCATCATGGCCAGGCCCGGAACTAATGCAAGACCCGCCTGAAG  3420

pBAD_CshA  3421  AGGGGACAGTCCCACAATCAACTGCAGATGCTGGGTAACGAAGTACAGGAAGCCCACTAA  3480
                 ||||||||||||||||||||||||||||||||||||||||||||||||||||||||||||
pZX_CshA   3421  AGGGGACAGTCCCACAATCAACTGCAGATGCTGGGTAACGAAGTACAGGAAGCCCACTAA  3480

pBAD_CshA  3481  GGCAACCACGCTCAGTAAGTTCACCAGCAGCGCTCCGGAAAAGGTGCCGCGGGAGAACAA  3540
                 ||||||||||||||||||||||||||||||||||||||||||||||||||||||||||||
pZX_CshA   3481  GGCAACCACGCTCAGTAAGTTCACCAGCAGCGCTCCGGAAAAGGTGCCGCGGGAGAACAA  3540

pBAD_CshA  3541  CCGCATATCCAGCATGGGCTCATTCACGCGGAGTTGACGGCGAACGAACAGCCAGCCAAA  3600
                 ||||||||||||||||||||||||||||||||||||||||||||||||||||||||||||
pZX_CshA   3541  CCGCATATCCAGCATGGGCTCATTCACGCGGAGTTGACGGCGAACGAACAGCCAGCCAAA  3600

pBAD_CshA  3601  CACCAGCCCCAACGCAAACAGGACCGGTGGGGTAATACCCCAGCCTTTCACTGCAAAGTC  3660
                 ||||||||||||||||||||||||||||||||||||||||||||||||||||||||||||
pZX_CshA   3601  CACCAGCCCCAACGCAAACAGGACCGGTGGGGTAATACCCCAGCCTTTCACTGCAAAGTC  3660

pBAD_CshA  3661  CTTGATCGCGTAGACAATGGGCACCATCGTCAGCATTGACAGCGCAATGCTCACCGGATC  3720
                 ||||||||||||||||||||||||||||||||||||||||||||||||||||||||||||
pZX_CshA   3661  CTTGATCGCGTAGACAATGGGCACCATCGTCAGCATTGACAGCGCAATGCTCACCGGATC  3720

pBAD_CshA  3721  AATGCGACCCGGACGCGGATCACGCGATTCTGGGACCAGAAACGGGGCCAGGACCAAAAG  3780
                 ||||||||||||||||||||||||||||||||||||||||||||||||||||||||||||
pZX_CshA   3721  AATGCGACCCGGACGCGGATCACGCGATTCTGGGACCAGAAACGGGGCCAGGACCAAAAG  3780

pBAD_CshA  3781  GGGCACCAGCACCGGTACCGCCATCAGGAACACAGAGCCCCAGCTGAAGTGTTCAAGCAG  3840
                 ||||||||||||||||||||||||||||||||||||||||||||||||||||||||||||
pZX_CshA   3781  GGGCACCAGCACCGGTACCGCCATCAGGAACACAGAGCCCCAGCTGAAGTGTTCAAGCAG  3840

pBAD_CshA  3841  CACCCCGCCGACAATTGGGCCCAGCGCCGCACCGGCAGAAAACATCGACGCCCAAACCGC  3900
                 ||||||||||||||||||||||||||||||||||||||||||||||||||||||||||||
pZX_CshA   3841  CACCCCGCCGACAATTGGGCCCAGCGCCGCACCGGCAGAAAACATCGACGCCCAAACCGC  3900

pBAD_CshA  3901  GATGGCGAGGCGACGCTGATCGCGGTCAGTGAAGATCGAGCGCAGCAGTGACAGGGTAGA  3960
                 ||||||||||||||||||||||||||||||||||||||||||||||||||||||||||||
pZX_CshA   3901  GATGGCGAGGCGACGCTGATCGCGGTCAGTGAAGATCGAGCGCAGCAGTGACAGGGTAGA  3960

pBAD_CshA  3961  GGGCATCAGCATCGCGCCGAACACGCCCATGGCGGCACGCGCTGCAATCAACAGTTCGGC  4020
                 ||||||||||||||||||||||||||||||||||||||||||||||||||||||||||||
pZX_CshA   3961  GGGCATCAGCATCGCGCCGAACACGCCCATGGCGGCACGCGCTGCAATCAACAGTTCGGC  4020

pBAD_CshA  4021  TGACGGTGCAAACGCTGCGAGCGCACTAACCGTTGCAAAACCGGTCGCGCCAATAAGTAA  4080
                 ||||||||||||||||||||||||||||||||||||||||||||||||||||||||||||
pZX_CshA   4021  TGACGGTGCAAACGCTGCGAGCGCACTAACCGTTGCAAAACCGGTCGCGCCAATAAGTAA  4080

pBAD_CshA  4081  CATGCGACGGCGGCCAAACCGATCACCCAGCGACCCCATAGTGACGAGCAGACCAGCCAG  4140
                 ||||||||||||||||||||||||||||||||||||||||||||||||||||||||||||
pZX_CshA   4081  CATGCGACGGCGGCCAAACCGATCACCCAGCGACCCCATAGTGACGAGCAGACCAGCCAG  4140
```

```
pBAD_CshA  4141  GATCAACGGATACACGTCGATAATCCAGAGCTGTTGGGCGGATGTAGGGCTTAACGCACG  4200
                 ||||||||||||||||||||||||||||||||||||||||||||||||||||||||||||
pZX_CshA   4141  GATCAACGGATACACGTCGATAATCCAGAGCTGTTGGGCGGATGTAGGGCTTAACGCACG  4200

pBAD_CshA  4201  CGCAATCTCCGGTAACGCAAAGCTTAAGACGGTGTTATCAACAGACACCAGGAGGACAGG  4260
                 ||||||||||||||||||||||||||||||||||||||||||||||||||||||||||||
pZX_CshA   4201  CGCAATCTCCGGTAACGCAAAGCTTAAGACGGTGTTATCAACAGACACCAGGAGGACAGG  4260

pBAD_CshA  4261  CAGCATGAGCACGCCTAAGGCTGCCCACCCACGCCAACCAACACGCCGGCCTTGACCATC  4320
                 ||||||||||||||||||||||||||||||||||||||||||||||||||||||||||||
pZX_CshA   4261  CAGCATGAGCACGCCTAAGGCTGCCCACCCACGCCAACCAACACGCCGGCCTTGACCATC  4320

pBAD_CshA  4321  AGCAATGGCGATCTCTTCCGTCAGAGTCATGGTGAATTCCTCCTGCTAGCCCAAAAAAAC  4380
                 ||||||||||||||||||||||||||||||||||||||||||||||||||||||||||||
pZX_CshA   4321  AGCAATGGCGATCTCTTCCGTCAGAGTCATGGTGAATTCCTCCTGCTAGCCCAAAAAAAC  4380

pBAD_CshA  4381  GGGTATGGAGAAACAGTAGAGAGTTGCGATAAAAAGCGTCAGGTAGGATCCGCTAATCTT  4440
                 ||||||||||||||||||||||||||||||||||||||||||||||||||||||||||||
pZX_CshA   4381  GGGTATGGAGAAACAGTAGAGAGTTGCGATAAAAAGCGTCAGGTAGGATCCGCTAATCTT  4440

pBAD_CshA  4441  ATGGATAAAAATGCTATGGCATAGCAAAGTGTGACGCCGTGCAAATAATCAATGTGGACT  4500
                 ||||||||||||||||||||||||||||||||||||||||||||||||||||||||||||
pZX_CshA   4441  ATGGATAAAAATGCTATGGCATAGCAAAGTGTGACGCCGTGCAAATAATCAATGTGGACT  4500

pBAD_CshA  4501  TTTCTGCCGTGATTATAGACACTTTTGTTACGCGTTTTTGTCATGGCTTTGGTCCCGCTT  4560
                 ||||||||||||||||||||||||||||||||||||||||||||||||||||||||||||
pZX_CshA   4501  TTTCTGCCGTGATTATAGACACTTTTGTTACGCGTTTTTGTCATGGCTTTGGTCCCGCTT  4560

pBAD_CshA  4561  TGTTACAGAATGCTTTTAATAAGCGGGGTTACCGGTTTGGTTAGCGAGAAGAGCCAGTAA  4620
                 ||||||||||||||||||||||||||||||||||||||||||||||||||||||||||||
pZX_CshA   4561  TGTTACAGAATGCTTTTAATAAGCGGGGTTACCGGTTTGGTTAGCGAGAAGAGCCAGTAA  4620

pBAD_CshA  4621  AAGACGCAGTGACGGCAATGTCTGATGCAATATGGACAATTGGTTTCTTCTCTGAATGGC  4680
                 ||||||||||||||||||||||||||||||||||||||||||||||||||||||||||||
pZX_CshA   4621  AAGACGCAGTGACGGCAATGTCTGATGCAATATGGACAATTGGTTTCTTCTCTGAATGGC  4680

pBAD_CshA  4681  GGGAGTATGAAAAGTATGGCTGAAGCGCAAAATGATCCCCTGCTGCCGGGATACTCGTTT  4740
                 ||||||||||||||||||||||||||||||||||||||||||||||||||||||||||||
pZX_CshA   4681  GGGAGTATGAAAAGTATGGCTGAAGCGCAAAATGATCCCCTGCTGCCGGGATACTCGTTT  4740

pBAD_CshA  4741  AATGCCCATCTGGTGGCGGGTTTAACGCCGATTGAGGCCAACGGTTATCTCGATTTTTTT  4800
                 ||||||||||||||||||||||||||||||||||||||||||||||||||||||||||||
pZX_CshA   4741  AATGCCCATCTGGTGGCGGGTTTAACGCCGATTGAGGCCAACGGTTATCTCGATTTTTTT  4800

pBAD_CshA  4801  ATCGACCGACCGCTGGGAATGAAAGGTTATATTCTCAATCTCACCATTCGCGGTCAGGGG  4860
                 ||||||||||||||||||||||||||||||||||||||||||||||||||||||||||||
pZX_CshA   4801  ATCGACCGACCGCTGGGAATGAAAGGTTATATTCTCAATCTCACCATTCGCGGTCAGGGG  4860

pBAD_CshA  4861  GTGGTGAAAAATCAGGGACGAGAATTTGTTTGCCGACCGGGTGATATTTTGCTGTTCCCG  4920
                 ||||||||||||||||||||||||||||||||||||||||||||||||||||||||||||
pZX_CshA   4861  GTGGTGAAAAATCAGGGACGAGAATTTGTTTGCCGACCGGGTGATATTTTGCTGTTCCCG  4920

pBAD_CshA  4921  CCAGGAGAGATTCATCACTACGGTCGTCATCCGGAGGCTCGCGAATGGTATCACCAGTGG  4980
                 ||||||||||||||||||||||||||||||||||||||||||||||||||||||||||||
pZX_CshA   4921  CCAGGAGAGATTCATCACTACGGTCGTCATCCGGAGGCTCGCGAATGGTATCACCAGTGG  4980
```

```
pBAD_CshA  4981  GTTTACTTTCGTCCGCGCGCCTACTGGCATGAATGGCTTAACTGGCCGTCAATATTTGCC  5040
                 ||||||||||||||||||||||||||||||||||||||||||||||||||||||||||||
pZX_CshA   4981  GTTTACTTTCGTCCGCGCGCCTACTGGCATGAATGGCTTAACTGGCCGTCAATATTTGCC  5040

pBAD_CshA  5041  AATACGGGGTTCTTTCGCCCGGATGAAGCGCACCAGCCGCATTTCAGCGACCTGTTTGGG  5100
                 ||||||||||||||||||||||||||||||||||||||||||||||||||||||||||||
pZX_CshA   5041  AATACGGGGTTCTTTCGCCCGGATGAAGCGCACCAGCCGCATTTCAGCGACCTGTTTGGG  5100

pBAD_CshA  5101  CAAATCATTAACGCCGGGCAAGGGGAAGGGCGCTATTCGGAGCTGCTGGCGATAAATCTG  5160
                 ||||||||||||||||||||||||||||||||||||||||||||||||||||||||||||
pZX_CshA   5101  CAAATCATTAACGCCGGGCAAGGGGAAGGGCGCTATTCGGAGCTGCTGGCGATAAATCTG  5160

pBAD_CshA  5161  CTTGAGCAATTGTTACTGCGGCGCATGGAAGCGATTAACGAGTCGCTCCATCCACCGATG  5220
                 ||||||||||||||||||||||||||||||||||||||||||||||||||||||||||||
pZX_CshA   5161  CTTGAGCAATTGTTACTGCGGCGCATGGAAGCGATTAACGAGTCGCTCCATCCACCGATG  5220

pBAD_CshA  5221  GATAATCGGGTACGCGAGGCTTGTCAGTACATCAGCGATCACCTGGCAGACAGCAATTTT  5280
                 ||||||||||||||||||||||||||||||||||||||||||||||||||||||||||||
pZX_CshA   5221  GATAATCGGGTACGCGAGGCTTGTCAGTACATCAGCGATCACCTGGCAGACAGCAATTTT  5280

pBAD_CshA  5281  GATATCGCCAGCGTCGCACAGCATGTTTGCTTGTCGCCGTCGCGTCTGTCACATCTTTTC  5340
                 ||||||||||||||||||||||||||||||||||||||||||||||||||||||||||||
pZX_CshA   5281  GATATCGCCAGCGTCGCACAGCATGTTTGCTTGTCGCCGTCGCGTCTGTCACATCTTTTC  5340

pBAD_CshA  5341  CGCCAGCAGTTAGGGATTAGCGTCTTAAGCTGGCGCGAGGACCAACGTATCAGCCAGGCG  5400
                 ||||||||||||||||||||||||||||||||||||||||||||||||||||||||||||
pZX_CshA   5341  CGCCAGCAGTTAGGGATTAGCGTCTTAAGCTGGCGCGAGGACCAACGTATCAGCCAGGCG  5400

pBAD_CshA  5401  AAGCTGCTTTTGAGCACCACCCGGATGCCTATCGCCACCGTCGGTCGCAATGTTGGTTTT  5460
                 ||||||||||||||||||||||||||||||||||||||||||||||||||||||||||||
pZX_CshA   5401  AAGCTGCTTTTGAGCACCACCCGGATGCCTATCGCCACCGTCGGTCGCAATGTTGGTTTT  5460

pBAD_CshA  5461  GACGATCAACTCTATTTCTCGCGGGTATTTAAAAAATGCACCGGGGCCAGCCCGAGCGAG  5520
                 ||||||||||||||||||||||||||||||||||||||||||||||||||||||||||||
pZX_CshA   5461  GACGATCAACTCTATTTCTCGCGGGTATTTAAAAAATGCACCGGGGCCAGCCCGAGCGAG  5520

pBAD_CshA  5521  TTCCGTGCCGGTTGTGAAGAAAAAGTGAATGATGTAGCCGTCAAGTTGTCATAATTGGTA  5580
                 ||||||||||||||||||||||||||||||||||||||||||||||||||||||||||||
pZX_CshA   5521  TTCCGTGCCGGTTGTGAAGAAAAAGTGAATGATGTAGCCGTCAAGTTGTCATAATTGGTA  5580

pBAD_CshA  5581  ACGAATCAGACAATTGACGGCTTGACGGAGTAGCATAGGGTTTGCAGAATCCCTGCTTCG  5640
                 ||||||||||||||||||||||||||||||||||||||||||||||||||||||||||||
pZX_CshA   5581  ACGAATCAGACAATTGACGGCTTGACGGAGTAGCATAGGGTTTGCAGAATCCCTGCTTCG  5640

pBAD_CshA  5641  TCCATTTGACAGGCACATTATGCATCGATGATAAGCTGTCAAACATGAGCAGATCCTCTA  5700
                 ||||||||||||||||||||||||||||||||||||||||||||||||||||||||||||
pZX_CshA   5641  TCCATTTGACAGGCACATTATGCATCGATGATAAGCTGTCAAACATGAGCAGATCCTCTA  5700

pBAD_CshA  5701  CGCCGGACGCATCGTGGCCGGCATCACCGGCGCCACAGGTGCGGTTGCTGGCGCCTATAT  5760
                 ||||||||||||||||||||||||||||||||||||||||||||||||||||||||||||
pZX_CshA   5701  CGCCGGACGCATCGTGGCCGGCATCACCGGCGCCACAGGTGCGGTTGCTGGCGCCTATAT  5760

pBAD_CshA  5761  CGCCGACATCACCGATGGGGAAGATCGGGCTCGCCACTTCGGGCTCATGAGCGCTTGTTT  5820
                 ||||||||||||||||||||||||||||||||||||||||||||||||||||||||||||
pZX_CshA   5761  CGCCGACATCACCGATGGGGAAGATCGGGCTCGCCACTTCGGGCTCATGAGCGCTTGTTT  5820
```

```
pBAD_CshA  5821  CGGCGTGGGTATGGTGGCAGGCCCCGTGGCCGGGGGACTGTTGGGCGCCATCTCCTTCTG  5880
                 ||||||||||||||||||||||||||||||||||||||||||||||||||||||||||||
pZX_CshA   5821  CGGCGTGGGTATGGTGGCAGGCCCCGTGGCCGGGGGACTGTTGGGCGCCATCTCCTTCTG  5880

pBAD_CshA  5881  CCTCGCGCGTTTCGGTGATGACGGTGAAAACCTCTGACACATGCAGCTCCCGGAGACGGT  5940
                 ||||||||||||||||||||||||||||||||||||||||||||||||||||||||||||
pZX_CshA   5881  CCTCGCGCGTTTCGGTGATGACGGTGAAAACCTCTGACACATGCAGCTCCCGGAGACGGT  5940

pBAD_CshA  5941  CACAGCTTGTCTGTAAGCGGATGCCGGGAGCAGACAAGCCCGTCAGGGCGCGTCAGCGGG  6000
                 ||||||||||||||||||||||||||||||||||||||||||||||||||||||||||||
pZX_CshA   5941  CACAGCTTGTCTGTAAGCGGATGCCGGGAGCAGACAAGCCCGTCAGGGCGCGTCAGCGGG  6000

pBAD_CshA  6001  TGTTGGCGGGTGTCGGGGCGCAGCCATGACCCTATGCGGTGTGAAATACCGCACAGATGC  6060
                 ||||||||||||||||||||||||||||||||||||||||||||||||||||||||||||
pZX_CshA   6001  TGTTGGCGGGTGTCGGGGCGCAGCCATGACCCTATGCGGTGTGAAATACCGCACAGATGC  6060

pBAD_CshA  6061  GTAAGGAGAAAATACCGCATCAGGCGC  6087
                 |||||||||||||||||||||||||||
pZX_CshA   6061  GTAAGGAGAAAATACCGCATCAGGCGC  6087
```

Supplementary Figure S1. Results of alignment analysis of the base sequences of pBAD_CshA and pZX_CshA using Blast2 sequence program (https://blast.ncbi.nlm.nih.gov/Blast.cgi?BLAST_SPEC=blast2seq&LINK_LOC=align2seq&PAGE_TYPE=BlastSearch).
